# Efficient Automatic Design of a 2D TMD FET via Machine Learning-Assisted TCAD Simulation

**DOI:** 10.3390/mi17080987

**Published:** 2026-08-21

**Authors:** Na Shi, Zi-Jun Wei, Tong Wu

**Affiliations:** 1 School of Microelectronics, Shanghai University, Shanghai 200444, China; shina@shu.edu.cn (N.S.); weizijun0110@shu.edu.cn (Z.-J.W.); 2 School of Integrated Circuits, Nanjing University, Suzhou 215163, China

**Keywords:** 2D TMD FET, TCAD simulation, machine learning, small-sample modeling, inverse design

## Abstract

As the scaling of silicon-based devices approaches physical limits, two-dimensional transition-metal dichalcogenide field-effect transistors (2D TMD FETs) have emerged as promising candidates for logic devices in the post-Moore era. However, their design optimization relies heavily on computationally intensive TCAD simulations, thereby limiting efficient exploration of multidimensional parameter spaces. This paper proposes an efficient automated design framework for 2D TMD FETs under small-sample conditions and validates it using a monolayer MoS_2_ FET as a case study. The framework integrates device design, physics-based simulation, performance prediction, and inverse design, establishing a bidirectional mapping between device parameters and electrical performance. Target-driven closed-loop optimization is achieved through TCAD-based feedback validation. Results demonstrate that, using a dataset comprising 300 TCAD samples, the forward model achieves an average coefficient of determination ($R^{2}$) of 0.9503. TCAD revalidation of the inverse-designed devices yields an average mean absolute error (MAE) of 0.0464 and an average mean absolute percentage error (MAPE) of 5.46% for performance metrics. Regarding computational efficiency, while a single TCAD simulation takes approximately 25 to 50 min, the trained model performs inference in under 50 ms, achieving a speedup of at least $3\times 10^{4}$ during the inference phase. Accounting for the generation of the 300 TCAD samples and the training of both forward and inverse models, the framework’s one-time computational cost ranges from 160.27 to 285.27 h. Once the cumulative number of design tasks exceeds approximately 342 to 385, the total computational cost falls below that of direct TCAD simulation, with the computational advantage becoming increasingly significant as the number of tasks grows. Consequently, this method is highly suitable for large-scale parameter sweeps, device screening, and multi-objective, high-frequency design iterations. It drastically reduces repetitive TCAD calls, offering a scalable solution for the efficient, automated design of 2D TMD FETs.

## 1. Introduction

As the cornerstone of modern integrated circuits, silicon-based metal oxide semiconductor field effect transistors (MOS FETs) have driven the development of the entire semiconductor industry through continuous scaling. However, according to the International Roadmap for Devices and Systems (IRDS), the scaling of silicon-based MOS FETs is approaching the physical limits of the material system [1]. To overcome this bottleneck, researchers have turned their attention to transition-metal dichalcogenides (TMDs), such as MoS_2_ and WS_2_ [2,3,4]. Two-dimensional transition-metal dichalcogenide field-effect transistors (2D TMD FETs) have achieved excellent electrostatic control capabilities due to their atomic-level thickness and excellent interface characteristics, showing significant advantages in suppressing short-channel effects, reducing power consumption, and improving switching speed [5,6,7]. In recent years, numerous theoretical and experimental studies have demonstrated that 2D TMD FETs can achieve high drive currents, low subthreshold swings, and fast switching speeds, even at gate lengths below 10 nm. These characteristics make 2D TMD FETs promising candidates for meeting the IRDS performance requirements for post-Moore logic devices. However, owing to their atomically thin channels, the electrical performance of these devices is highly sensitive to the material band structure, metal–semiconductor contact properties, and surrounding dielectric environment. Moreover, these physical factors are strongly and nonlinearly coupled, substantially increasing the complexity of device design and optimization. Technology computer-aided design (TCAD) simulation has therefore become an essential tool for analyzing these coupled physical effects, evaluating device characteristics, and guiding structural optimization. Although TCAD can provide physically accurate device characteristics, its computational cost becomes prohibitive when applied to multidimensional parameter co-optimization, where the number of required simulations increases rapidly with the dimensionality of the design space.

Machine learning (ML) provides a new approach to addressing the above challenges [8,9]. The main idea is to build an ML-based forward model. This model learns the mapping between device structural parameters and electrical performance from TCAD-generated data. Therefore, it can replace expensive and time-consuming TCAD simulation while maintaining high prediction accuracy within the sampled design space. This data-driven approach can predict device performance in real time and quickly scan parameters. Theoretically, it can shorten the design cycle by several orders of magnitude. Numerous studies have confirmed the effectiveness of ML techniques in accelerating semiconductor device design and optimization [10,11,12].

However, existing studies still have several notable limitations. First, most previous works have focused on improving the predictive accuracy of surrogate models using large-scale datasets [13,14], while insufficient attention has been paid to small-sample modeling when TCAD data acquisition is constrained [15]. In 2D TMD FET design, the high computational cost and long runtime of each TCAD simulation severely limit the amount of available training data. Consequently, conventional machine learning models are prone to unstable predictions and insufficient generalization under small-sample conditions. Second, systematic methods for evaluating the robustness of small-sample surrogate models remain limited. The reliability of model predictions and their consistency with the underlying device physics have not been systematically investigated, thereby restricting their practical application [16]. In addition, current machine learning-assisted TCAD studies primarily focus on the forward problem of predicting electrical performance from device structural parameters, whereas inverse design, which directly derives optimal device parameters from specified target performance, remains insufficiently explored [17]. Existing inverse-design methods generally rely on iterative search strategies, such as genetic algorithms, particle swarm optimization, and differential evolution [18]. These methods are not only computationally inefficient but also struggle to achieve stable and scalable optimization in high-dimensional parameter spaces.

To address these research gaps, this paper proposes an efficient automated design framework for 2D TMD FETs under small-sample conditions. The S-cGAN regression method is adapted to small-sample modeling and incorporated into a closed-loop forward–inverse design workflow, which is validated using a monolayer MoS_2_ FET as a case study. Compared with existing machine learning-assisted device-optimization methods, the proposed framework differs in three main aspects. First, to address the high cost of TCAD data acquisition, the proposed model is developed using only 300 TCAD samples rather than relying on a large-scale training dataset. Second, in addition to predictive accuracy, bootstrap analysis and SHapley Additive exPlanations (SHAP) analysis are introduced to evaluate the statistical robustness and physical interpretability of the model, respectively, thereby improving the credibility of small-sample modeling. Finally, the framework is not limited to the unidirectional mapping from device parameters to electrical performance. It further establishes an inverse generative model that maps target performance to device parameters, and the generated parameters are subsequently revalidated using TCAD simulations. This establishes a complete closed-loop workflow encompassing device design, TCAD simulation, performance prediction, inverse generation, and physical-consistency validation.

**Figure 1:**
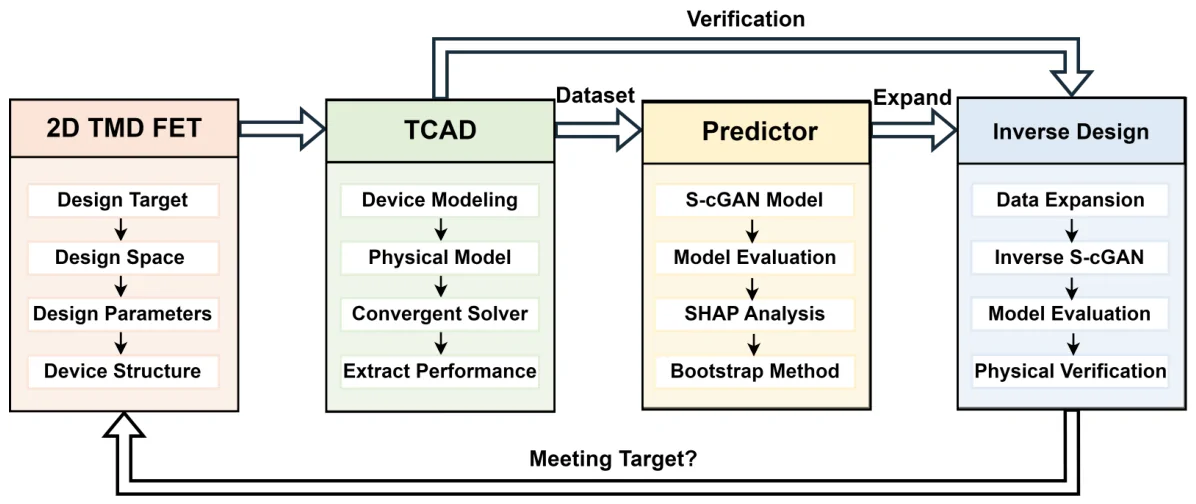
An efficient automated design framework for ML-assisted TCAD simulation under small-sample conditions.

Figure 1 illustrates the proposed forward–inverse co-design workflow and the data flow among its constituent modules. First, the device design space and structural parameters are defined according to the design objectives. An experimentally calibrated TCAD model is then employed for device modeling, physics-based simulation, and electrical-performance extraction, thereby constructing a reliable dataset. Subsequently, this dataset is used to train the S-cGAN performance-prediction model, which establishes a fast mapping from device parameters to electrical performance. The predictive accuracy, interpretability, and robustness of the model are evaluated using performance metrics, SHapley Additive exPlanations (SHAP) analysis, and bootstrap analysis, respectively.

On this basis, the performance-prediction model is used to expand the dataset, which is subsequently employed to train the inverse S-cGAN model. The trained inverse model can directly generate candidate device parameters according to specified target-performance requirements. Finally, the inversely generated parameters are fed back into the TCAD model for physics-based validation. If the resulting performance fails to satisfy the design targets, the design is further refined through the feedback loop until device parameters meeting the target requirements are obtained. This process forms a closed-loop design workflow encompassing device design, TCAD simulation, forward prediction, inverse generation, and physics-based validation. The main contributions of this work are summarized as follows:

1. A high-accuracy TCAD physical model for a monolayer MoS_2_ FET is established. Within the drift–diffusion transport framework, the model comprehensively incorporates key physical mechanisms, including quantum corrections, mobility degradation, contact resistance, interface traps, and fixed charges. It is jointly calibrated using multiple sets of experimental transfer-characteristic curves measured at different channel lengths, enabling an accurate description of the principal electrical behavior of the device.
2. An S-cGAN surrogate model suitable for small-sample scenarios is proposed. Using only 300 samples, the model achieves an accurate mapping from seven-dimensional device parameters to 25-dimensional electrical responses, with an average coefficient of determination ($R^{2}$) of 0.9503. Bootstrap-based robustness analysis and SHapley Additive exPlanations (SHAP) parameter-sensitivity analysis are further incorporated to enhance model robustness and decision interpretability.
3. A data-driven inverse-design module is developed to directly generate device parameters from target-performance specifications, thereby avoiding the large number of TCAD calls required by conventional iterative optimization methods. The results show that after the parameters generated by inverse design are validated through TCAD simulations, the resulting performance exhibits an average mean absolute error (MAE) of 0.0464 and an average mean absolute percentage error (MAPE) of 5.46% relative to the target performance.
4. A complete workflow for the efficient design of 2D TMD FETs is constructed, forming a closed design loop that integrates device design, TCAD simulation, performance prediction, inverse parameter generation, and physical-consistency validation.

The remainder of this paper is organized as follows. Section 2 describes the device structure and physical modeling. Section 3 develops an ML-based performance prediction model for small-sample datasets. It also presents a target-performance-oriented inverse design method and realizes forward and inverse co-design. Section 4 systematically analyzes and validates the proposed method. The analysis includes the robustness of the performance prediction model under small-sample conditions and the physical consistency of the inversely designed device parameters in TCAD simulations. Section 5 concludes this paper. It summarizes the advantages of the proposed method in improving the design efficiency of 2D TMD FETs.

## 2. Device Structure and Physical Modeling

### 2.1. Monolayer MoS_2_ FET Device Structure

To overcome the scaling limitations of silicon-based complementary metal oxide semiconductor (CMOS) technology, FETs based on monolayer MoS_2_ channels have been considered promising candidates for next-generation logic devices owing to their atomic-scale thickness and strong electrostatic control [19]. However, conventional metal contacts can induce metal-induced gap states (MIGS) at the metal/MoS_2_ interface, resulting in strong Fermi-level pinning and the formation of a substantial Schottky barrier. These interfacial effects impede carrier injection and substantially increase the contact resistance [20]. To mitigate these limitations, semimetallic Sb is employed in this work to establish a van der Waals (vdW) contact with monolayer MoS_2_, thereby suppressing interfacial gap states and facilitating low-resistance carrier injection [21].

**Figure 2:**
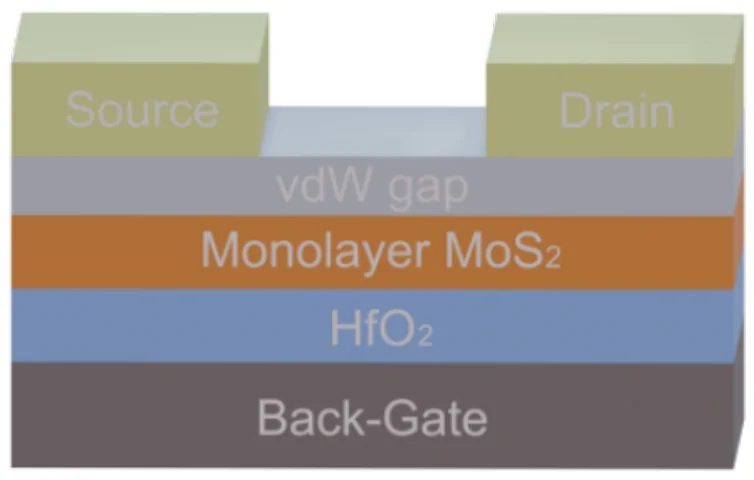
Monolayer MoS_2_ FET device structure (not to scale).

As illustrated in Figure 2, the schematic structure of the monolayer MoS_2_ FET is presented. The source and drain contact electrodes use a stacked metal structure of 30-nm Au/20-nm Sb. Semimetallic Sb is used to form a vdW contact interface with MoS_2_. This interface is dominated by weak vdW interactions. An atomically flat vdW gap is formed between Sb and MoS_2_. This gap can suppress the generation of MIGS. It also reduces the height and width of the Schottky barrier. As a result, the probability of electron tunneling injection is enhanced. A degenerately doped region is formed near the contact interface, which enables low-resistance Ohmic contact. The high-κ metal gate stack consists of 4-nm Ti/30-nm Au/8.5-nm HfO_2_.

### 2.2. TCAD Simulations

In this section, a physics-based TCAD simulation model is built for the monolayer MoS_2_ FET and systematically calibrated using experimental data. Within the drift-diffusion transport framework, the model incorporates key physical models such as quantum correction, mobility degradation, contact resistance, interface traps, and fixed charge. To accurately describe carrier transport, interface effects, and contact properties in the monolayer MoS_2_ FET, the simulation model parameters are also calibrated. It can therefore reproduce the key electrical behavior of the device.

The simulations were performed using Sentaurus TCAD 2022.03. In Sentaurus Structure Editor (SDE), local nanoscale mesh refinement was applied to the MoS_2_ channel and contact-gap regions. The numerical solution employed the Super direct solver together with a fully coupled Newton iteration scheme. In addition, 128-bit extended precision and a stringent residual convergence criterion of $10^{-8}$ were adopted to improve numerical accuracy and stability. Regarding the physical models, Wentzel–Kramers–Brillouin (WKB) tunneling in the source/drain contact regions was calculated using a nonlocal tunneling model. The interface traps at the HfO_2_/MoS_2_ interface were described using a superposition of uniform and Gaussian energy distributions of acceptor-like states, and the Trap Damped Line-Search Newton (TrapDLN) method was employed to improve the numerical stability of the trap-charge calculation. The physical model is configured as follows:

1. Mobility model: A high-field saturation model and an interface mobility degradation model are used. By comprehensively considering factors such as phonon scattering, interface scattering, Coulomb scattering, and high-field velocity saturation, this model takes into account both low-field and high-field transport characteristics. It can simultaneously and accurately describe the intrinsic transport of long-channel devices and the current degradation behavior of short-channel devices under strong electric fields and interface scattering.
2. Charge model: A density-functional-theory (DFT) calibrated multivalley density-of-states (DoS) model is adopted. First-principles calculation results are introduced to parameterize and correct the intrinsic band structure of the material. Key physical parameters, such as the bandgap, carrier effective mass, and effective DoS, are accurately extracted. This model fully considers the contribution of different energy valleys to carrier transport and can accurately describe the band dispersion relation and carrier distribution behavior of MoS_2_.
3. Contact-resistance model: For the contact between semimetallic Sb and MoS_2_, a barrier-tunneling model based on the vdW gap and the WKB approximation is employed [22]. The vdW gap at the Sb/MoS_2_ interface is treated as an effective tunneling barrier. The energy-dependent tunneling probability is evaluated from the spatial barrier profile using the WKB approximation, and the corresponding nonlocal tunneling current is calculated to model carrier injection and contact resistance.
4. Interface-trap and fixed-charge model: At the MoS_2_/HfO_2_ interface, an interface trap density $D_{\mathrm{it}}$ with a tunable energy distribution and fixed positive charges $Q_{f}$ are introduced. They are used to reproduce the experimental subthreshold swing, threshold voltage, and flat-band voltage. Thus, the effects of interface charges and scattering on the device can be accurately described. Here, $D_{\mathrm{it}}$ is modeled by the superposition of a uniform distribution and a Gaussian distribution. The uniform component describes background defect states, while the Gaussian component describes localized deep-level defects [23,24]. In addition, $Q_{f}$ is used to represent non-exchangeable positive charge centers at the interface.

**Figure 3:**
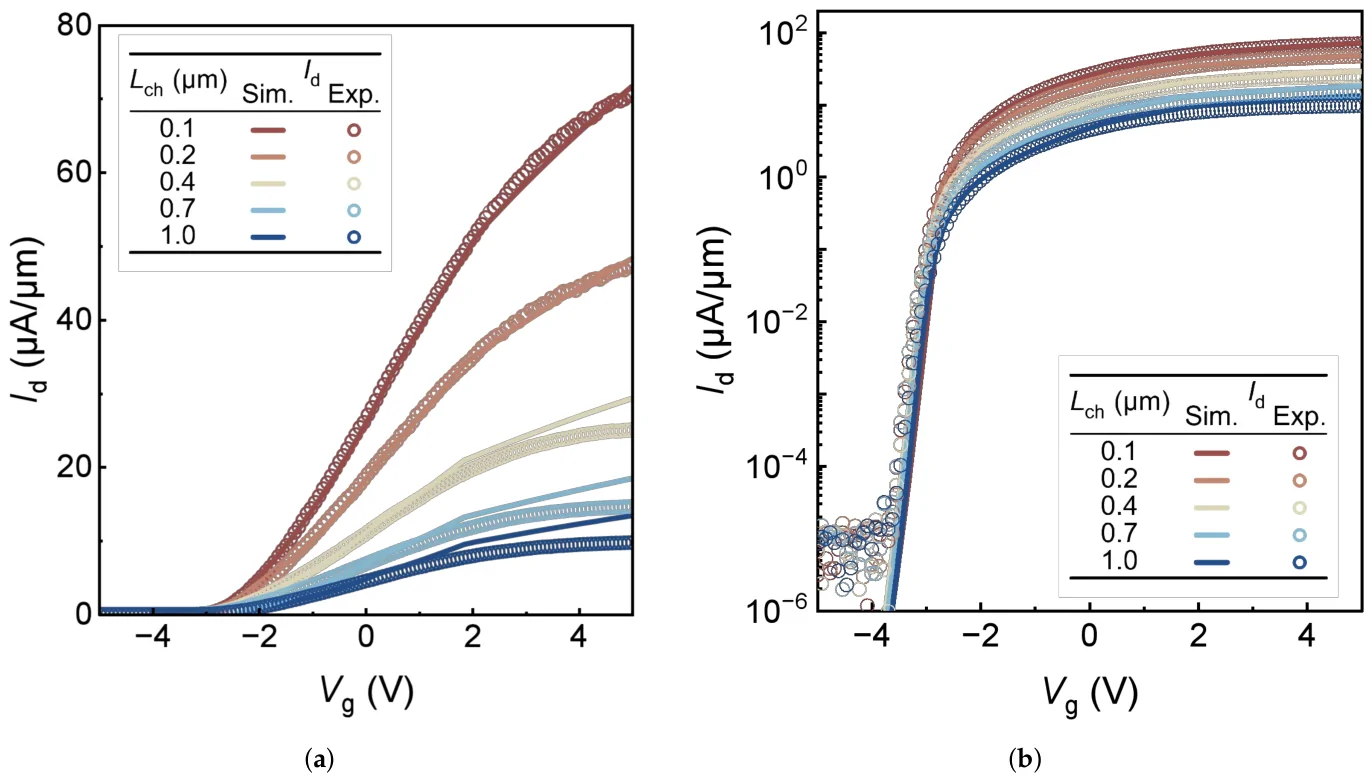
Simulated and experimental $I_{d}$–$V_{g}$ characteristics of monolayer MoS_2_ FETs with channel lengths of 0.1, 0.2, 0.4, 0.7, and 1.0 μm at $V_{d}=50\;\mathrm{mV}$. (**a**) $I_{d}$–$V_{g}$ characteristics on a linear scale. (**b**) $I_{d}$–$V_{g}$ characteristics on a logarithmic scale.

The TCAD model parameters are calibrated using experimental transfer characteristics measured at different channel lengths. As shown in Figure 3, the calibrated simulation results agree well with the experimental data over the entire gate-voltage range, confirming the accuracy and reliability of the established model. This work does not freely fit a large number of parameters using a single $I_{d}$–$V_{g}$ curve. Instead, physics-constrained joint calibration across multiple performance regimes, device dimensions, and operating conditions is employed to reduce the likelihood of parameter degeneracy. The current calibration focuses on the low-drain-voltage regime (the linear region), which is primarily used to extract key static metrics such as the threshold voltage and subthreshold swing. This focus is consistent with the optimization objectives of this work, although it provides relatively weaker constraints on parameters associated with high-field transport and thermal effects. In future work, output characteristics measured at different drain biases and temperature-dependent experimental data will be incorporated to enable more comprehensive validation of high-field transport, mobility degradation, and thermal effects.

The calibrated TCAD model parameters are summarized in Table 1. These fixed baseline parameters describe the material properties, process conditions, and physical-model settings used for calibration, enabling the model to capture the key electrical characteristics and dominant physical mechanisms of the monolayer MoS_2_ FET. Based on the calibrated model, Table 2 lists the key tunable design parameters and their physically reasonable ranges, including $L_{\mathrm{ch}}$, $t_{\mathrm{ox}}$, $W_{m}$, $N_{\mathrm{imp}}$, $\varepsilon_{r}$, μ, and $D_{\mathrm{it}}$. Among these parameters, $L_{\mathrm{ch}}$ and $t_{\mathrm{ox}}$ are typical device structural parameters. $W_{m}$ and $N_{\mathrm{imp}}$ are primarily process-controllable parameters: $W_{m}$ can be adjusted through gate-metal selection and interface engineering, whereas $N_{\mathrm{imp}}$ can be controlled through processes such as doping and surface charge transfer. $\varepsilon_{r}$, μ, and $D_{\mathrm{it}}$ are mainly introduced into the TCAD model as effective material or physical parameters. The range of μ is based on the effective channel mobility extracted from the calibrated TCAD model and represents typical values for the back-gated devices considered in this work under practical operating conditions at room temperature. These values are used as the initial mobility inputs for TCAD design-parameter sweeps and should not be interpreted as the theoretical mobility at the phonon-scattering limit. The prescribed lower and upper bounds define a physically constrained parameter space and provide clear search boundaries for the subsequent target-driven inverse design. Four key metrics are extracted to describe the transport characteristics: threshold voltage $V_{\mathrm{th}}$, subthreshold swing (SS), on-state current $I_{\mathrm{on}}$, and off-state current $I_{\mathrm{off}}$.

The gate voltage at $I_{d}=10^{-7}$ A is defined as $V_{\mathrm{th}}$. This definition has a clear physical meaning. It can also be used to compare the performance of different devices. SS is used to evaluate the gate-control capability in the subthreshold region. It is extracted using the transconductance-based method and is given by

(1) $$\mathrm{SS}={\left(\frac{d(\log_{10}I_{d})}{dV_{g}}\right)}^{-1}.$$

This value is the inverse slope of the $I_{d}$–$V_{g}$ curve in the subthreshold region on a logarithmic scale. The on-state current $I_{\mathrm{on}}$ and off-state current $I_{\mathrm{off}}$ describe the driving capability and the turn-off behavior of the device, respectively. Their ratio,

(2) $$\mathrm{Ratio}=\frac{I_{\mathrm{on}}}{I_{\mathrm{off}}},$$

reflects the switching performance of the device. In addition, $I_{\mathrm{off}}$ is closely related to the static power consumption. The static power is calculated as

(3) $$P_{\mathrm{static}}=I_{\mathrm{off}}V_{d}.$$

## 3. Machine Learning for 2D TMD FET Design

**Figure 4:**
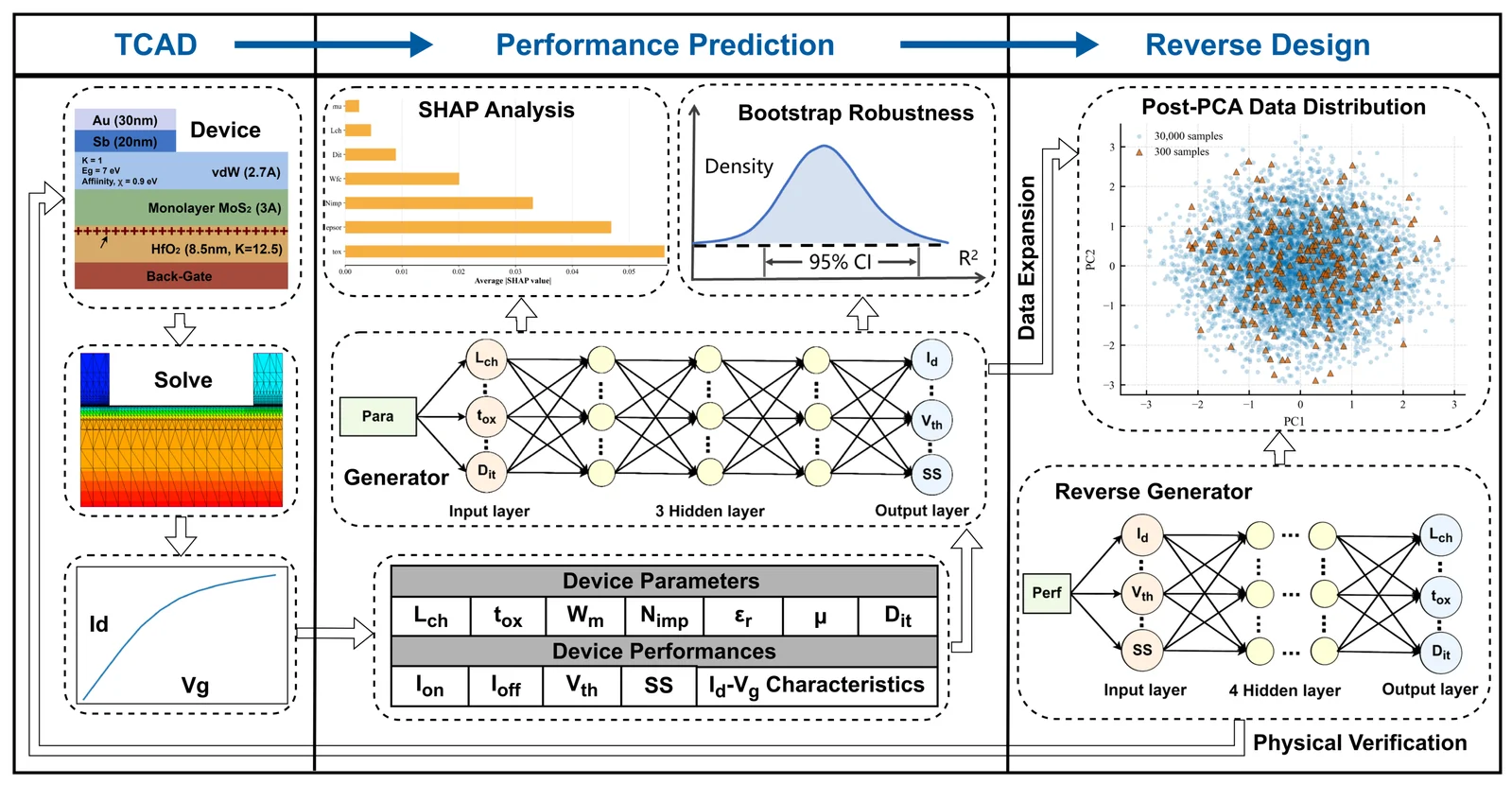
A small-sample forward and inverse co-design workflow for monolayer MoS_2_ FETs based on S-cGAN.

In this section, the design optimization of a monolayer MoS_2_ FET is used as an example. An efficient automated design framework for 2D TMD FETs under small-sample conditions is proposed. The detailed implementation flow of the framework is shown in Figure 4. First, under the design target constraints, training data are obtained by TCAD simulations based on an accurate physical model. Then, a predictor is used to establish an accurate mapping from device structural parameters to electrical performance. This mapping accelerates the TCAD simulation process. Finally, an inverse design module is developed to directly generate device parameters from target performance specifications. The generated parameters are further verified by TCAD simulations.

It is worth noting that the proposed framework is specifically developed for small-sample scenarios. By integrating bootstrap-based robustness analysis with SHapley Additive exPlanations (SHAP) parameter-sensitivity analysis, it significantly enhances the model’s robustness and the interpretability of its predictions.

### 3.1. Dataset Construction and Pre-Processing

To ensure the generalization capability of the forward model under small-sample conditions, a random sampling strategy is used to construct the dataset. Specifically, 300 n-type monolayer MoS_2_ FET device instances are first randomly generated in the predefined parameter design space using the Monte Carlo method. The design space consists of seven parameters, including $L_{\mathrm{ch}}$, $t_{\mathrm{ox}}$, $W_{m}$, $N_{\mathrm{imp}}$, $\varepsilon_{r}$, μ, and $D_{\mathrm{it}}$. The range of each parameter is listed in Table 2. Then, for each device instance, the calibrated TCAD model is used to simulate the electrical characteristics. At a fixed drain voltage, the gate voltage $V_{g}$ is swept from −8V to 2V with a step size of 0.25V. A total of 41 drain-current data points are extracted to construct the $I_{d}$–$V_{g}$ transfer characteristic curve, from which key electrical metrics, including $I_{\mathrm{on}}$, $I_{\mathrm{off}}$, $V_{\mathrm{th}}$, and SS, are further obtained.

Based on the above procedure, a dataset with 300 samples is constructed. To obtain reliable performance evaluation under limited data conditions, five-fold cross-validation is adopted, with an 8:2 train–test split. The input data consist of seven-dimensional device parameters, whereas the output data consist of 25-dimensional device-performance responses. The output vector contains four scalar performance metrics, namely $I_{\mathrm{on}}$, $I_{\mathrm{off}}$, $V_{\mathrm{th}}$, and SS, together with 21 drain-current values sampled from the $I_{d}$–$V_{g}$ transfer curve. The complete 25-dimensional performance vector is used as the conditioning target for inverse design. In this representation, the four scalar metrics characterize the key switching properties of the device, while the 21 drain-current values preserve the overall transfer characteristics and help maintain physical consistency across the entire operating range. The 21 current points are sampled from the original 41-point transfer curve at a gate-voltage interval of 0.5 V. This sampling strategy preserves the main features of the transfer characteristics while reducing the output dimensionality and simplifying the learning task of the forward model.

In the data pre-processing stage, Min–Max normalization is applied to each dimension of the input and output data. This step removes the influence of different physical units on model training. In addition, the current-related data span several orders of magnitude. Therefore, a logarithmic transformation is first applied to compress their dynamic range. The transformed data are then normalized. This pre-processing improves the stability and convergence of model training.

### 3.2. Small-Sample Performance Prediction Model

To achieve a fast and accurate mapping from device structural parameters to electrical performance, this work constructs an S-cGAN-based performance prediction model. A purely supervised regression model is prone to overfitting with 300 samples. Although a standard cGAN introduces adversarial training, its discriminator can also overfit under small-sample conditions, leading to unstable generator training. Based on cGAN, S-cGAN further adds supervised constraints and dual loss optimization. Specifically, the adversarial loss keeps the generator output distribution close to the TCAD simulation data. The reconstruction loss directly reduces the error between the predicted and true values at each output point. This greatly improves training stability and prediction accuracy. It is a key design choice for the small-sample, high-dimensional regression task in this work. It should be noted that the novelty of this work does not lie in proposing an entirely new S-cGAN architecture, but rather in adapting the S-cGAN regression method to device-design scenarios characterized by costly TCAD data acquisition and limited training samples and integrating it into a closed-loop forward–inverse design framework.

**Figure 5:**
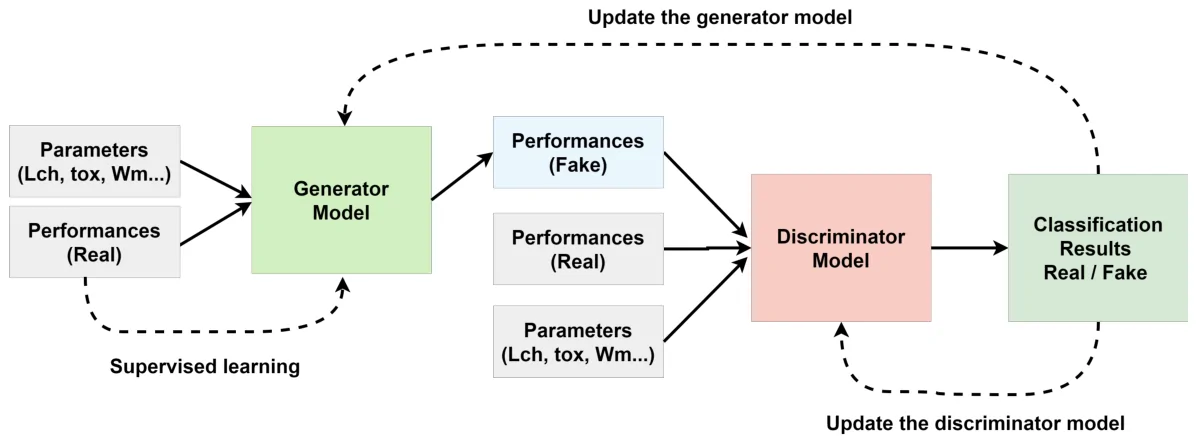
Framework diagram of the S-cGAN model used in this study.

As shown in Figure 5, the generator predicts electrical performance from the input device structural parameters, and the discriminator evaluates the consistency between the predicted and TCAD-simulated results. The two networks are optimized alternately, jointly driving the model to accurately capture the complex nonlinear relationship between structural parameters and electrical characteristics under limited data conditions.

The input of the generator consists of 7-dimensional device parameters and a 10-dimensional noise vector. The input is passed through three fully connected layers with 2048, 1024, and 512 neurons, respectively. Each layer is followed by a LeakyReLU activation function with a negative slope of 0.2 and BatchNorm1d normalization. Finally, a linear output layer maps the extracted features to a 25-dimensional space and outputs the device performance. The noise vector introduces small perturbations at the input side. This helps improve the generalization capability of the model. The input of the discriminator is the concatenation of the 25-dimensional device performance vector and the 7-dimensional device parameter vector. The device performance vector can be either the real performance data or the performance predicted by the generator. The concatenated input is then passed through two fully connected layers with 1024 and 512 neurons, respectively. Each hidden layer is also followed by a LeakyReLU activation function and BatchNorm1d normalization. Finally, a fully connected layer and a Sigmoid function output a discrimination probability between 0 and 1. A value of 0 denotes a predicted sample, while a value of 1 denotes a real sample.

The S-cGAN is trained in a mini-max manner, which is implemented by alternately minimizing the generator and discriminator losses. The generator is trained to produce performance vectors that are indistinguishable from the TCAD-simulated data. The discriminator is trained to maximize its ability to distinguish real samples from generated samples. In this work, the loss function of the generator consists of two parts: the adversarial loss and the reconstruction loss. The adversarial loss is based on the cross-entropy loss and describes the adversarial training between the generator and the discriminator. The reconstruction loss adopts the MAE, which provides supervised guidance for the generator by encouraging the generated performance vector to be close to the real one. The generator losses are defined as

(4) $$L_{G,\mathrm{adv}}=-E_{x,z}\left[\log D\left(G(x,z),x\right)\right],$$

(5) $$L_{G,\mathrm{recon}}=E_{x,y,z}\left[{∥y-G\left(x,z\right)∥}_{1}\right],$$

(6) $$\begin{array}{c} L_{G}=L_{G,\mathrm{adv}}+\lambda_{\mathrm{recon}}L_{G,\mathrm{recon}}, \end{array}$$

where x denotes the 7-dimensional device parameter vector, y denotes the 25-dimensional real device performance vector and z denotes the 10-dimensional noise vector, which follows the standard normal distribution N(0,1). G(x,z) is the predicted performance generated by the generator. D(G(x,z),x) denotes the probability predicted by the discriminator that the parameter–performance pair is real. The weight $\lambda_{\mathrm{recon}}$ is set to 10 to balance adversarial learning and supervised regression.

Unlike the generator loss, which is used to optimize the generator *G*, the discriminator loss is used to optimize the discriminator *D*. The discriminator is trained to distinguish real performance samples from generated ones. Its loss function consists of two terms. The first term corresponds to the real-sample classification loss, which encourages the discriminator to assign a high probability to the real pair (y,x). The second term corresponds to the generated-sample classification loss, which encourages the discriminator to assign a low probability to the generated pair (G(x,z),x). The discriminator losses are written as

(7) $$L_{D1}=-E_{x,y}\left[\log D(y,x)\right],$$

(8) $$L_{D2}=-E_{x,z}\left[\log\left(1-D(G(x,z),x)\right)\right],$$

(9) $$\begin{array}{c} L_{D}=L_{D1}+L_{D2}. \end{array}$$

The model is trained using an alternating optimization strategy for adversarial learning. Learning-rate warmup, a cosine annealing scheduler, and gradient clipping are also introduced to improve training stability. The model is trained for 1000 epochs with a batch size of 64. For each batch, the real samples are first constructed using the real device parameter–performance pairs. The fake samples are constructed using the predicted performance generated by the generator. The discriminator is updated using the binary cross-entropy loss. This enables the discriminator to distinguish real performance data from generated performance data. Then, the discriminator is fixed. The generator produces performance responses from the input device parameters and random noise. Two losses are minimized at the same time. The first one is the adversarial loss, which encourages the generated performance vector to be indistinguishable from real TCAD data. The second one is the reconstruction loss, which makes the generated performance close to the real performance in value. Both the generator and the discriminator are optimized using the AdamW optimizer. The initial learning rate is set to 0.0001 for both networks. The momentum parameters are set to $\left(\beta_{1},\beta_{2}\right)=\left(0.5,0.999\right)$. The learning rate is linearly warmed up during the first 100 epochs. It is then scheduled using cosine annealing. The minimum learning rate is set to 0.1 times the initial learning rate. In addition, the gradient norm is clipped to be no larger than 5.0.

It should be noted that the forward-prediction task in this work is essentially a supervised regression problem, in which the generator of the S-cGAN performs the regression mapping from device parameters to electrical performance. Unlike a conventional artificial neural network (ANN), the S-cGAN further incorporates a conditional discriminator and an adversarial loss. Conventional supervised learning mainly employs the MAE or mean squared error (MSE) to constrain pointwise discrepancies between the predicted values and the TCAD results. In contrast, adversarial learning additionally constrains the correlations among multidimensional electrical responses from an overall distributional perspective. Consequently, the generated results are not only numerically close to the ground-truth values, but also exhibit a joint distribution that more closely resembles that of the original TCAD data [25,26]. Moreover, previous studies have demonstrated that continuous conditional generative approaches offer advantages in continuous regression tasks and scenarios with locally sparse samples [27]. Therefore, the supervised loss is employed to ensure prediction accuracy, whereas the adversarial loss serves as an auxiliary distribution-level constraint to enhance the modeling capability under limited TCAD data.

To improve the interpretability of the model, SHAP analysis is introduced in this work. It is used to analyze the feature contributions in the prediction mechanism of the generator. The main idea of SHAP is to decompose the model prediction into the contributions of all input features. SHAP values measure the average marginal change in the prediction when a feature is added to the model. The sum of the SHAP values of all features is equal to the change between the prediction and the baseline value. Therefore, SHAP can explain individual predictions and provide a global ranking of feature importance. In this work, a single-output prediction function is constructed for each performance dimension. The noise vector is fixed to zero to avoid the influence of random perturbations on the interpretation results. Then, KernelExplainer is used to calculate the SHAP values for each output dimension. In this way, the marginal contribution of each device parameter to different performance metrics can be obtained.

**Figure 6:**
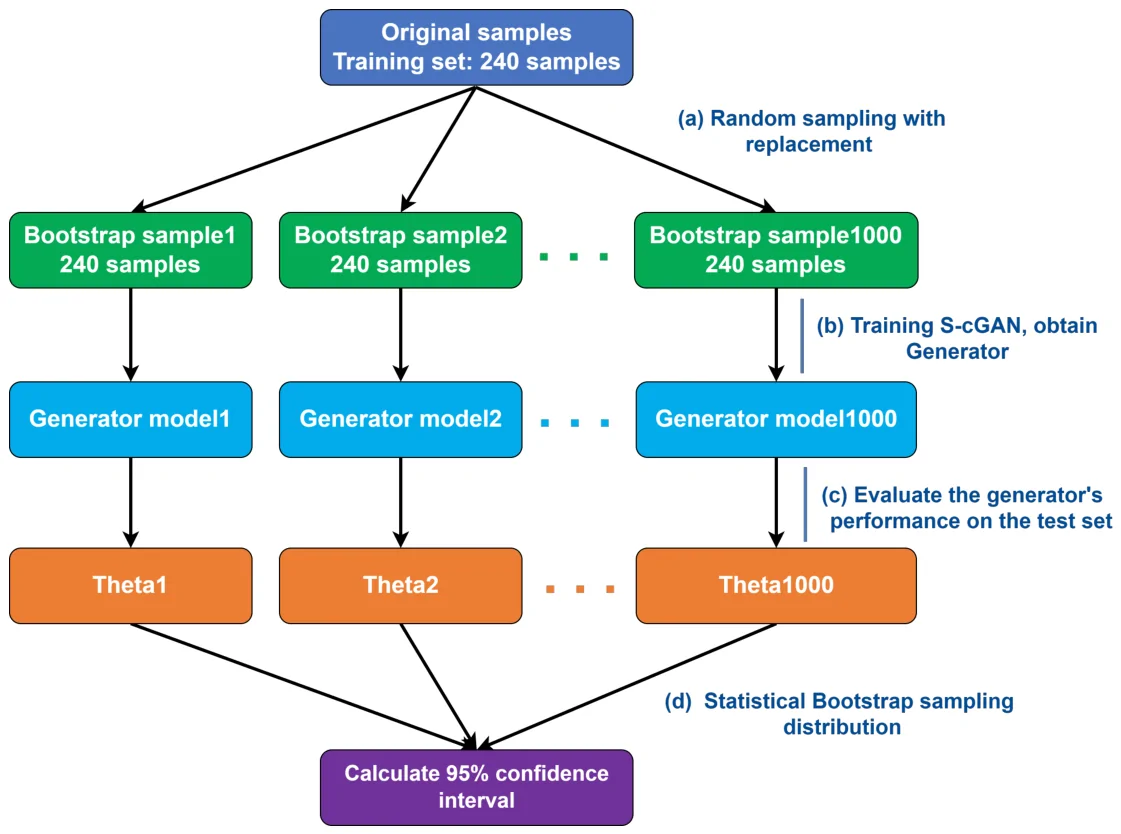
Implementation process of the bootstrap method. Theta is a collection of device performance evaluation metrics.

To evaluate the predictive stability of the model under small-sample conditions, a bootstrap-based robustness analysis is performed. By repeatedly resampling the original data with replacement, the bootstrap method simulates random fluctuations in the data distribution caused by the limited sample size and quantifies the uncertainty in model performance. It is a widely used and effective statistical evaluation method for small-sample analysis [28,29]. Figure 6 illustrates the implementation workflow of the bootstrap analysis. In each bootstrap iteration, 240 samples are drawn with replacement from the original training set of 240 samples to form a new bootstrap dataset, thereby representing a different possible composition of the limited training data. An S-cGAN forward model is then trained using this bootstrap dataset and evaluated on the same independent test set containing 60 previously unseen samples. Performance metrics, including the $R^{2}$ and MAE, are calculated to characterize the predictive accuracy of the model for the current bootstrap sample. This procedure is repeated 1000 times, producing 1000 sets of performance metrics that approximate the empirical distributions of model performance under different training-sample perturbations.

Kernel density estimation (KDE) is subsequently applied to the 1000 metric values to obtain smoothed probability-density distributions. The 2.5th and 97.5th percentiles of the corresponding cumulative distributions are used as the lower and upper endpoints of the 95% confidence intervals, respectively. These intervals characterize the variation in model performance caused by perturbations in the training samples. A narrower confidence interval indicates that the model is less sensitive to differences in the sampled training data and therefore exhibits more stable predictive performance. Accordingly, the resulting confidence intervals provide a quantitative measure of uncertainty in model performance and a more robust statistical basis for the practical application of the proposed model.

### 3.3. Inverse Design

The forward mapping can rapidly predict electrical performance when the device structural parameters are given. However, practical device design is more concerned with another problem. That is, how to determine the optimal structural parameters under given target performance constraints. Conventional iterative optimization methods, such as genetic algorithms and particle swarm optimization, usually need to call computationally intensive TCAD simulations at each iteration. This leads to a long design cycle. To address this issue, this work proposes a data-driven inverse design method. It aims to realize a fast mapping from target performance to structural parameters. Thus, the design iteration cycle can be greatly reduced.

Compared with the forward mapping problem, the inverse design problem is more complex. The same set of performance metrics may correspond to multiple sets of structural parameters. In addition, the parameter space is high-dimensional and strongly coupled. Therefore, training an inverse model only with the original 300 samples may cause underfitting and poor generalization. It is also difficult to obtain stable inverse prediction. To improve the learnability and coverage of the inverse model, the small-data performance prediction model built in the previous section is used for data augmentation. In the parameter design space, 30,000 structural-parameter samples are randomly generated. The previously developed performance prediction model is then used to replace TCAD simulations and obtain the corresponding key performance metrics. The generated structural parameters and predicted performance metrics constitute the augmented dataset for training the inverse design model.

For the inverse design model, the existing S-cGAN framework is modified to fit the inverse mapping task. Since the inverse design task is more complex, the network structures of the generator and discriminator are enhanced and optimized to improve model performance. The input of the generator is changed to the 25-dimensional device performance vector and a 10-dimensional noise vector. The input is passed through four fully connected layers with 1024, 1024, 512, and 256 neurons, respectively. These layers are used for feature extraction and mapping. In the first four hidden layers, each linear transformation is followed by BatchNorm1d and a LeakyReLU activation function with a negative slope of 0.2. Dropout layers are added after the first three hidden layers with dropout rates of 0.3, 0.3, and 0.2, respectively. This improves the regularization capability of the model. Finally, a fully connected output layer maps the features to the 7-dimensional parameter space. A Sigmoid activation function is used to constrain the output to the range of [0,1], which corresponds to the normalized device parameters.

The input to the discriminator remains the concatenation of a 25-dimensional device performance vector and a 7-dimensional device parameter vector. The parameter vector is obtained either from the real dataset or from the generator. Then, it passes through three fully connected layers with 512, 512, and 256 neurons. Each hidden layer uses a LeakyReLU activation function with a negative slope of 0.2. Dropout with a rate of 0.3 is applied after the first two layers to reduce overfitting. Finally, a fully connected layer followed by a Sigmoid activation function produces a probability between 0 and 1, indicating whether the input parameter vector is a real sample from the dataset or a synthetic sample generated by the generator.

It should be noted that the performance labels of the augmented data are generated by the forward surrogate model and therefore inevitably contain a certain degree of prediction error. In this work, these data are used only as auxiliary samples to increase the dataset size and improve the coverage of the training data for the inverse model. They are not used as the basis for the physical validation of the final design results. To prevent surrogate-model errors from propagating through the inverse-design process and affecting the final conclusions, a TCAD validation step is introduced after the inverse model completes its prediction. Specifically, the device parameters generated by the inverse model are fed back into the calibrated TCAD model for simulation, and the resulting TCAD-simulated electrical responses are compared with the target performance. Therefore, the final design results are evaluated using independent physics-based simulations rather than data generated by the forward surrogate model itself. By establishing a closed-loop workflow that combines data-driven parameter generation with TCAD-based physical validation, the proposed framework improves parameter-search efficiency while ensuring that the final design results are supported by independent physical simulations. This approach effectively mitigates the bias caused by surrogate-model self-reference and ensures the physical credibility of the inverse-designed devices.

## 4. Results and Discussion

### 4.1. Performance Analysis of Small Dataset Models

In the small-data S-cGAN-based performance prediction model, the generator plays the key role in mapping device structural parameters to electrical performance. Its prediction accuracy directly determines the effectiveness and reliability of the forward model under small-sample conditions. Therefore, we systematically evaluate the modeling capability of several common regression algorithms in the small-data scenario. This comparison is conducted as a standalone supervised forward-prediction task to justify the use of an artificial neural network (ANN) structure as the backbone of the S-cGAN generator. The compared baseline models include ANN, random forest (RF), gradient boosting regression (GB), ridge regression (Ridge), and support vector regression (SVR). It should be noted that RF, GB, Ridge, and SVR are used only as baseline forward models for performance comparison and are not embedded into the adversarial generator of the S-cGAN. The key parameter settings for each algorithm are shown in Table 3.

**Figure 7:**
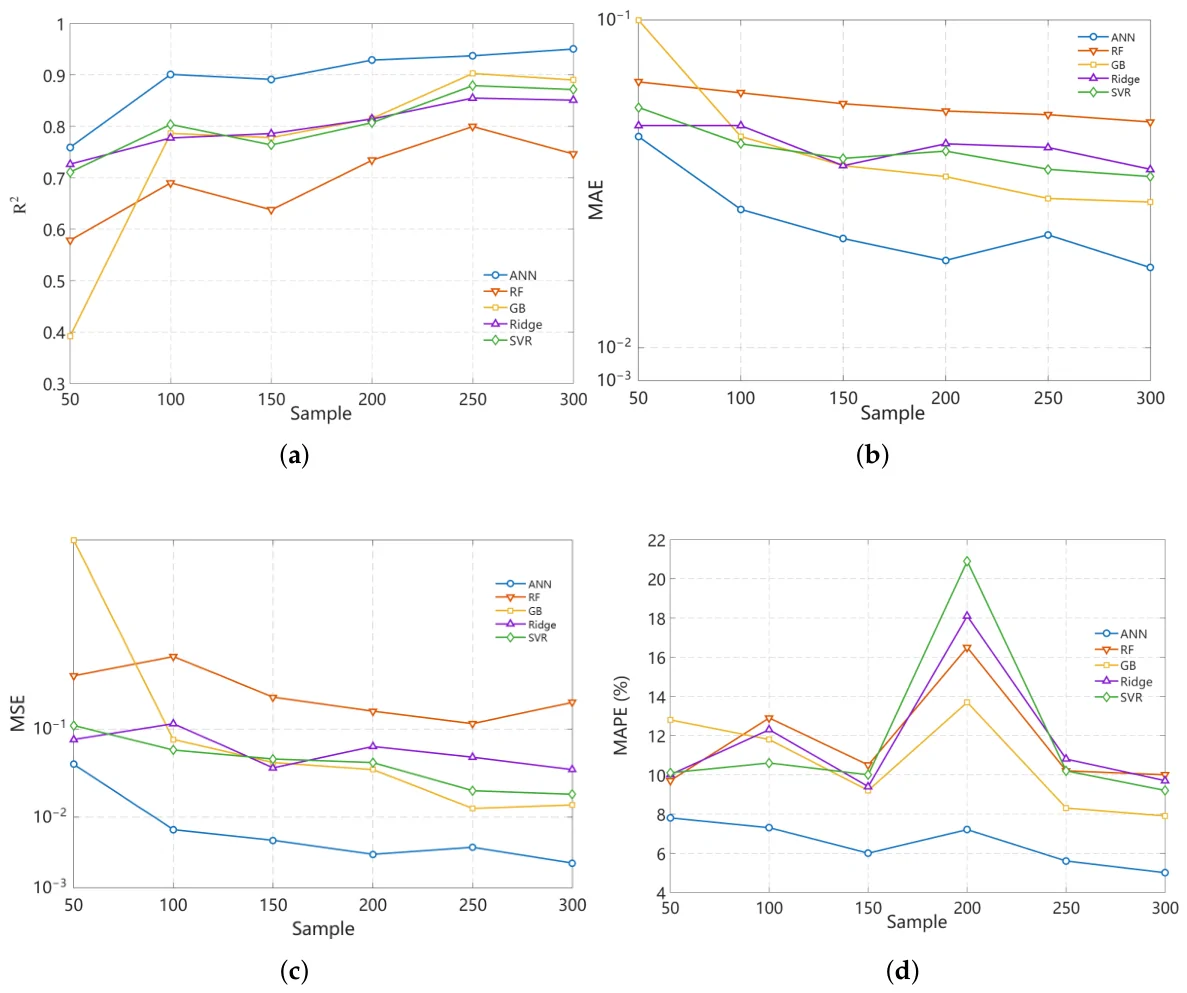
Averaged performance metrics of five algorithms for 25-dimensional outputs under different sample sizes. (**a**) $R^{2}$ scores. (**b**) MAE. (**c**) MSE. (**d**) MAPE.

Figure 7 shows the prediction performance of the five algorithms under different sample sizes. To comprehensively evaluate the model accuracy, four metrics are used for comparison, including $R^{2}$, MAE, MSE, and MAPE.

As shown in Figure 7, as the number of samples increases from 50 to 300, the overall prediction performance of each model improves, although the improvement is not strictly monotonic. Especially when the sample size is 200, most models exhibit significant performance fluctuations. In the MAPE metric, both the SVR and Ridge models show significant peaks in prediction error, which may be due to occasional shifts in the data distribution of this batch of samples or the presence of local noise anomalies, rather than a systematic decline in model performance. Among all the compared algorithms, ANN demonstrates the most stable and superior performance. Across different sample sizes, it achieves the highest average $R^{2}$ and maintains the lowest prediction errors, indicating stronger nonlinear mapping capability and generalization ability. Notably, ANN is relatively sensitive to increases in sample size, whereas RF, GB, SVR, and Ridge show more limited responses to changes in sample size. Their performance fluctuations are mainly caused by differences in data quality and sample distribution. This phenomenon further suggests that, under the modeling conditions of the S-cGAN framework for small-sample scenarios, data quality and distribution often have a more critical impact on model performance than changes in sample size.

**Figure 8:**
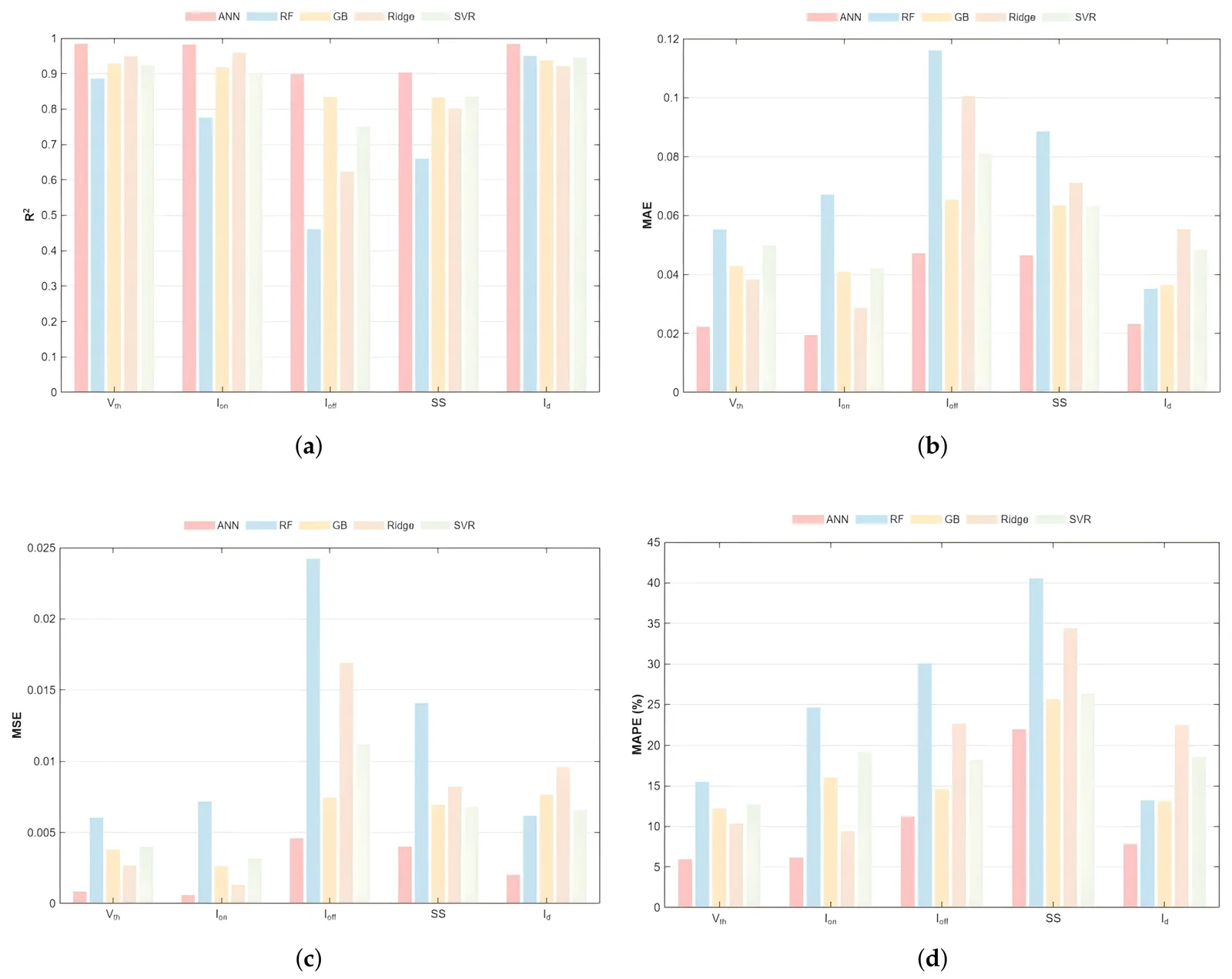
Performance comparison of five algorithms for predicting key electrical performances and drain-current responses. (**a**) $R^{2}$ scores. (**b**) MAE. (**c**) MSE. (**d**) MAPE.

Figure 8 further compares the prediction performance of different models on the key electrical metrics, including $I_{\mathrm{on}}$, $I_{\mathrm{off}}$, $V_{\mathrm{th}}$, and SS, as well as on the 21-dimensional drain current curves $I_{d}$. The results show that ANN achieves the best prediction performance for all metrics. This indicates that ANN can effectively learn the complex nonlinear mapping between device structural parameters and electrical responses. However, all models show relatively large prediction errors for $I_{\mathrm{off}}$ and SS. This is because $I_{\mathrm{off}}$ and SS are more sensitive to variations in device structure and process parameters. They also show stronger nonlinearity and data dispersion. In addition, the nonuniform distribution of SS data and the limited number of samples further increase the difficulty of learning its variation trend. For $I_{\mathrm{off}}$, the current level is usually very low. It is therefore more easily affected by changes in numerical scale and small parameter perturbations. This makes its prediction more difficult. Based on the comparison results of the five baseline models, the ANN architecture is adopted as the backbone of the S-cGAN generator because of its superior nonlinear mapping capability and stable prediction accuracy under small-sample conditions.

To further verify the practical contribution of adversarial learning, a discriminator ablation experiment was conducted. The discriminator was removed while keeping the dataset, generator architecture, and supervised loss unchanged, such that the artificial neural network (ANN) was trained using regression alone. As shown in Table 4, under the extremely limited-data conditions of 50 and 100 samples, the ANN achieved slightly higher $R^{2}$ values than the S-cGAN, indicating that adversarial training may introduce additional fluctuations when the number of samples is excessively small. When the sample size increased to 150 or more, the S-cGAN consistently outperformed the ANN. For sample sizes of 150, 200, 250, and 300, the average $R^{2}$ values increased by 0.0315, 0.0125, 0.0047, and 0.0034, respectively.

These results indicate that adversarial learning does not necessarily provide an advantage under all extremely small-sample conditions. However, once sufficient sample-space coverage is achieved, the distribution-level constraint provided by the discriminator can further improve the generalization capability of the model beyond supervised regression alone, thereby confirming the effectiveness of introducing the S-cGAN in this work.

Table 5 lists the prediction accuracy of the S-cGAN model with ANN as the generator. The results include the key electrical metrics, namely $I_{\mathrm{on}}$, $I_{\mathrm{off}}$, $V_{\mathrm{th}}$, and SS, as well as the 21-dimensional $I_{d}$–$V_{g}$ curve. The results show that the model achieves good overall prediction performance. The average $R^{2}$ reaches 0.9503, and all error metrics are within an acceptable range. It should be noted that $R^{2}$, MAE, and MSE are calculated on the normalized data scale. This ensures a consistent evaluation criterion among different output dimensions. In contrast, MAPE is used to measure the relative error. Therefore, it is calculated after inverse normalization to the original physical scale. Since the current values span several orders of magnitude, the MAPE values of $I_{\mathrm{off}}$ and the $I_{d}$–$V_{g}$ curve are calculated on the logarithmic scale. This avoids excessive amplification of the relative error caused by extremely small current values.

**Figure 9:**
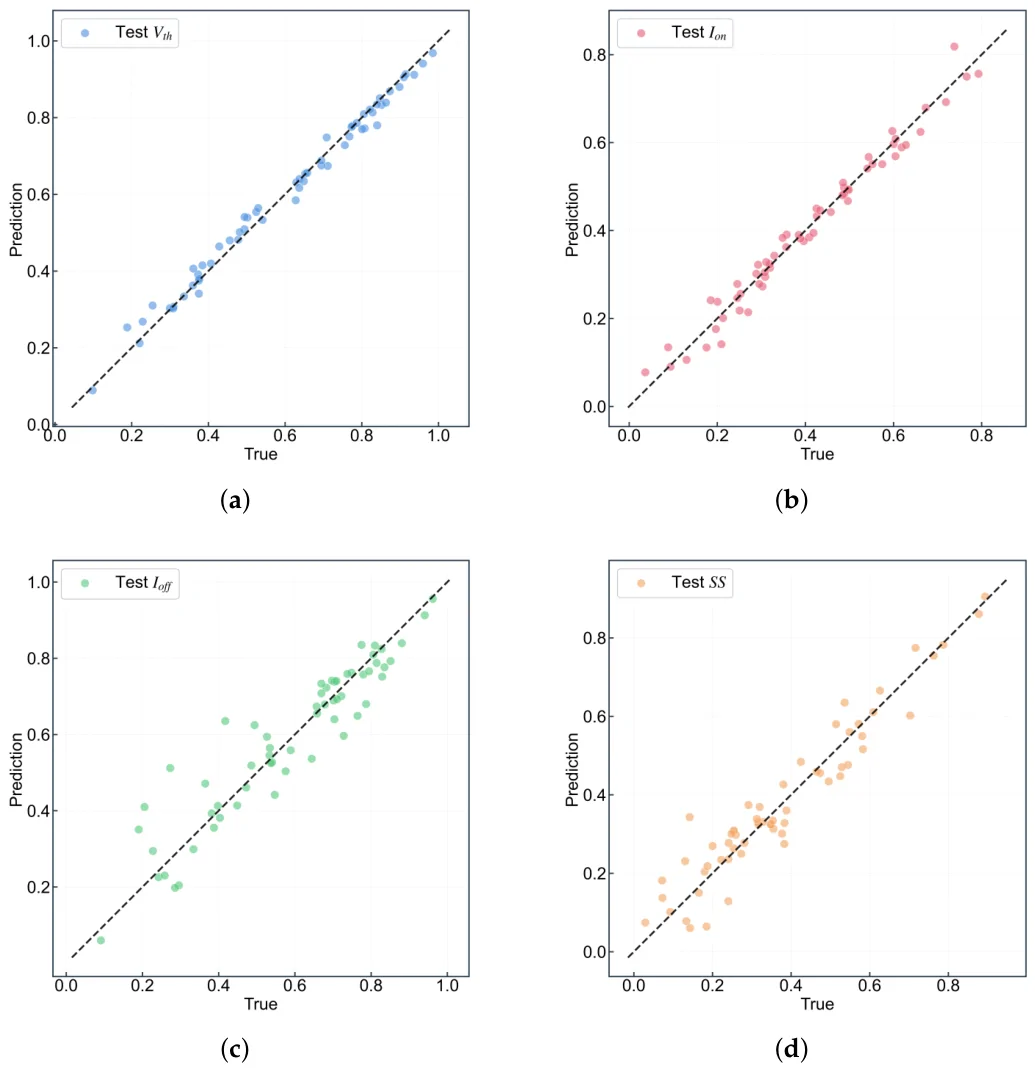
Scatter plots showing the predicted device performances by the S-cGAN model. (**a**) $V_{\mathrm{th}}$. (**b**) $I_{\mathrm{on}}$. (**c**) $I_{\mathrm{off}}$. (**d**) SS.

Figure 9 presents the agreement between the model-predicted key performance metrics and the TCAD simulation values using scatter plots. In addition to quantitative evaluation, the prediction capability of the model should also be further verified from the viewpoint of physical device characteristics. The $I_{d}$–$V_{g}$ curve is an important measure for evaluating the $V_{\mathrm{th}}$, $I_{\mathrm{on}}$, $I_{\mathrm{off}}$, and SS characteristics of monolayer MoS_2_ FETs. It is also important for device design and structural optimization.

**Figure 10:**
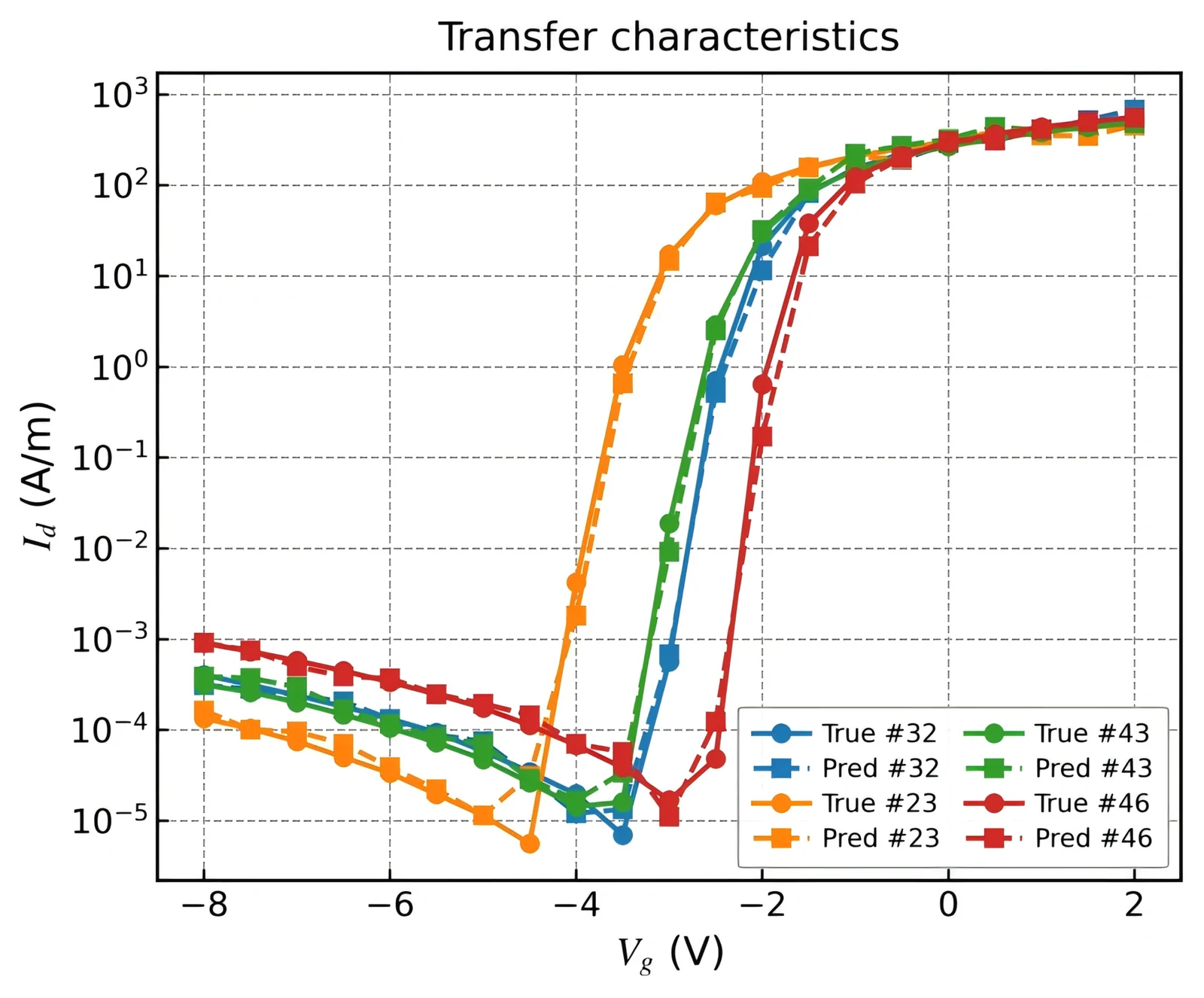
Transfer characteristics of monolayer MoS_2_ FETs.

Figure 10 clearly shows that the transfer characteristic curves obtained from TCAD simulations and model predictions are highly consistent across different samples. This indicates that the model can accurately capture the variation trend of the $I_{d}$–$V_{g}$ curves. These results show that the constructed forward model has good physical reliability. The model can effectively reproduce the physical transport behavior of the device.

**Figure 11:**
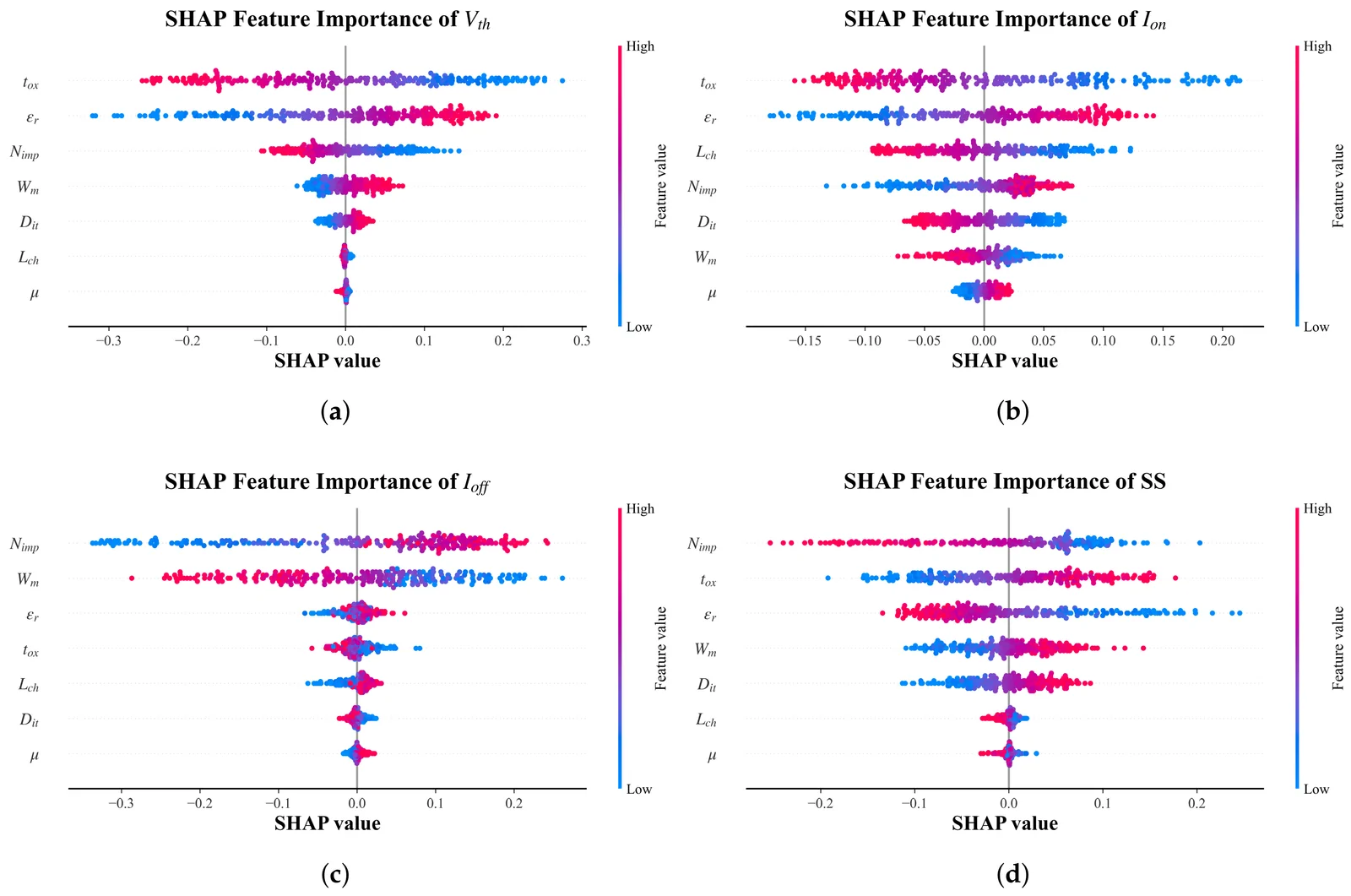
SHAP analysis results for key electrical performance indicators. (**a**) $V_{\mathrm{th}}$. (**b**) $I_{\mathrm{on}}$. (**c**) $I_{\mathrm{off}}$. (**d**) SS.

To further verify the physical interpretability of the model prediction results, SHAP analysis is introduced in this work. It is used to quantify the contribution of each design parameter to the key performance metrics. As shown in Figure 11, the horizontal axis represents the SHAP value. It indicates the positive or negative effect of a parameter on the prediction result. The color represents the magnitude of the parameter value. The vertical order reflects the importance of the parameters. The SHAP analysis results are highly consistent with the device physics. It should be noted that the low SHAP contribution of mobility is a relative result within the design range considered in this work and does not imply that mobility has no physical effect. Mobility is retained as an input parameter to ensure that the design space covers extreme cases, including low-mobility conditions, and that the generated device parameters remain consistent with realistic carrier-transport physics. The SHAP analysis provides a quantitative basis for evaluating the physical interpretability of the model and offers valuable guidance for prioritizing device design parameters and conducting subsequent inverse-design searches.

**Figure 12:**
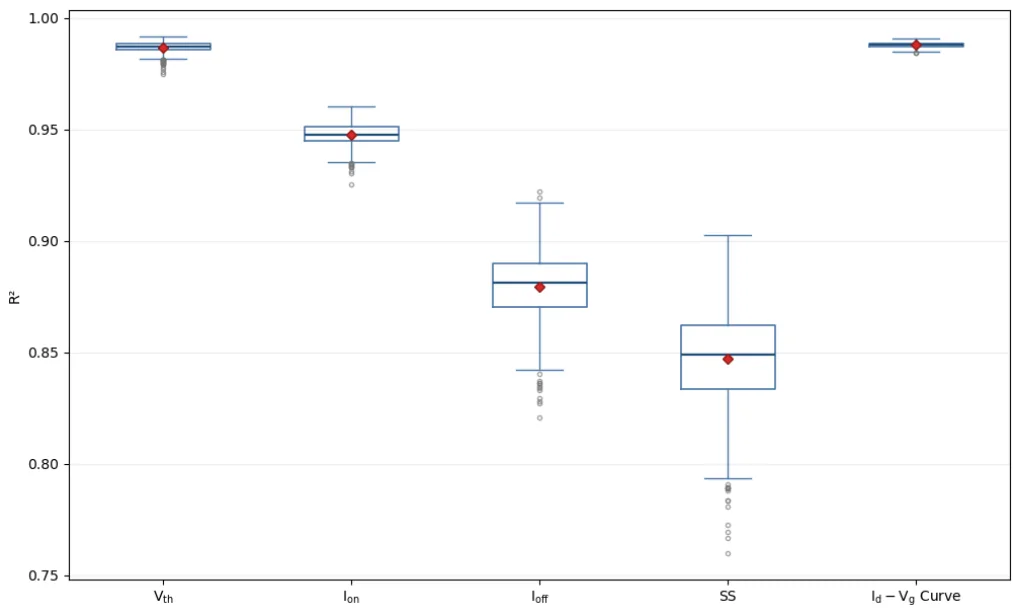
The box plots of $R^{2}$ from the 1000 bootstrap iterations.

The bootstrap method is employed to evaluate the robustness of the model under small-sample conditions. Specifically, the original set of 240 training samples is resampled with replacement 1000 times. After each resampling iteration, the surrogate model is retrained and evaluated, and the predictive accuracy of each performance metric on the test set is recorded to obtain its empirical distribution. Figure 12 presents box plots of the $R^{2}$ values obtained from the 1000 bootstrap iterations, while Table 6 summarizes the corresponding means, standard deviations, medians, and 95% confidence intervals.

The results show that $V_{\mathrm{th}}$ and the $I_{d}$–$V_{g}$ curves achieve the highest $R^{2}$ values and exhibit the smallest variations, indicating that the model provides stable and reliable predictions of the threshold voltage and transfer characteristics. The prediction accuracy for $I_{\mathrm{on}}$ is also relatively high, although it is slightly lower than those of the preceding two outputs. In contrast, the $R^{2}$ distributions of $I_{\mathrm{off}}$ and SS are broader and have lower mean values, indicating greater predictive uncertainty for these two metrics under small-sample conditions. This observation is consistent with the physical characteristics of $I_{\mathrm{off}}$ and SS, which are more sensitive to variations in device parameters and are more susceptible to local underfitting when the available samples provide insufficient coverage of the design space. Overall, the average $R^{2}$ is 0.930, with a standard deviation of only 0.006 and a 95% confidence interval of [0.918,0.940], demonstrating that the model exhibits good overall robustness under small-sample conditions. The prediction errors are primarily concentrated in subthreshold-related metrics, namely $I_{\mathrm{off}}$ and SS. By comparison, the model achieves higher prediction accuracy and smaller variations for $V_{\mathrm{th}}$, $I_{\mathrm{on}}$, and the $I_{d}$–$V_{g}$ curves, which are associated with the on-state or overall transfer characteristics.

Although 300 samples may be limited for the complex mapping from seven inputs to 25 outputs studied in this work, all samples follow the same device physics. They are not independent random observations but different results produced by the same physical mechanism. Therefore, model training aims to learn and represent this mechanism, rather than simply fit the data statistically. The training data were generated by TCAD simulations calibrated with experimental results. Physical rules were used to limit the design parameter space, and the S-cGAN uses the TCAD simulation results as the target for the reconstruction loss. In this way, prior knowledge of device physics is directly included in the training process. Previous studies have shown that adding physical simulation rules or prior physical knowledge to model training can provide high prediction accuracy even with limited training data and can reduce the risk of overfitting in small datasets [30,31]. In addition, the bootstrap robustness analysis presented in Figure 12 and Table 6 show that, after 1000 resampling iterations, the model achieves a mean $R^{2}$ of 0.930 with a standard deviation of only 0.006. The resulting variance is very small, indicating that the reported high $R^{2}$ is not an incidental consequence of overfitting but rather reflects the model’s stable capture of the underlying device physics.

**Figure 13:**
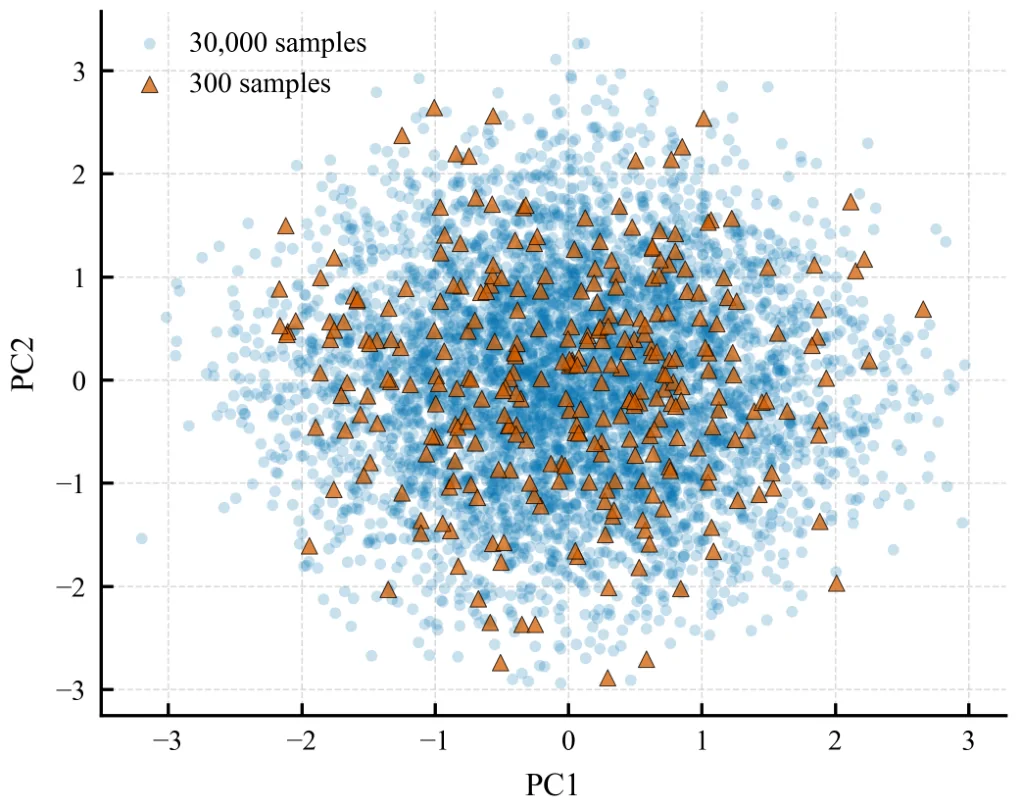
Distribution of 300 original samples and 30,000 expanded samples in the 7-dimensional parameter space after PCA dimensionality reduction.

To further evaluate the representativeness of the random-sampling strategy in the seven-dimensional design space, principal component analysis (PCA) was performed exclusively on the seven standardized device-design parameters. The analysis compares the 300 original samples with the 30,000 augmented samples used for inverse design, as shown in Figure 13. The first two principal components jointly explain approximately 29% of the total variance, with PC1 and PC2 accounting for 14.49% and 14.47%, respectively. The distribution of the 300 samples largely overlaps with the data cloud formed by the 30,000 augmented samples. It covers the densely populated central region and extends toward the boundary regions, with no large or systematic unexplored gaps being observed. Nevertheless, the sampling density of the 300 samples remains relatively limited in sparsely populated boundary regions, indicating that random sampling still has certain limitations near local boundaries. Overall, the Monte Carlo random-sampling strategy provides reasonably good spatial coverage of the current seven-dimensional design space. The subsequent five-fold cross-validation and bootstrap robustness analysis further support the robustness of the model-evaluation results under limited-sample conditions.

It should be emphasized that PCA can reveal only the overall coverage trend in a low-dimensional projection and cannot fully characterize potential local sampling gaps in the original high-dimensional space. Future work may therefore incorporate space-filling methods, such as Latin hypercube sampling (LHS), to further improve the uniformity and representativeness of sampling in high-dimensional design spaces.

### 4.2. Inverse Design to Obtain Optimal Parameters

The inverse design problem is inherently more complex because of its “one-to-many” nature, which means that the same set of performance metrics may correspond to multiple sets of device structural parameters. To address this issue, the previously developed performance prediction model is used to expand the dataset to 30,000 samples in the parameter space. Based on the augmented dataset, the inverse S-cGAN model is trained to achieve direct mapping and generation from target performance to device structural parameters.

**Figure 14:**
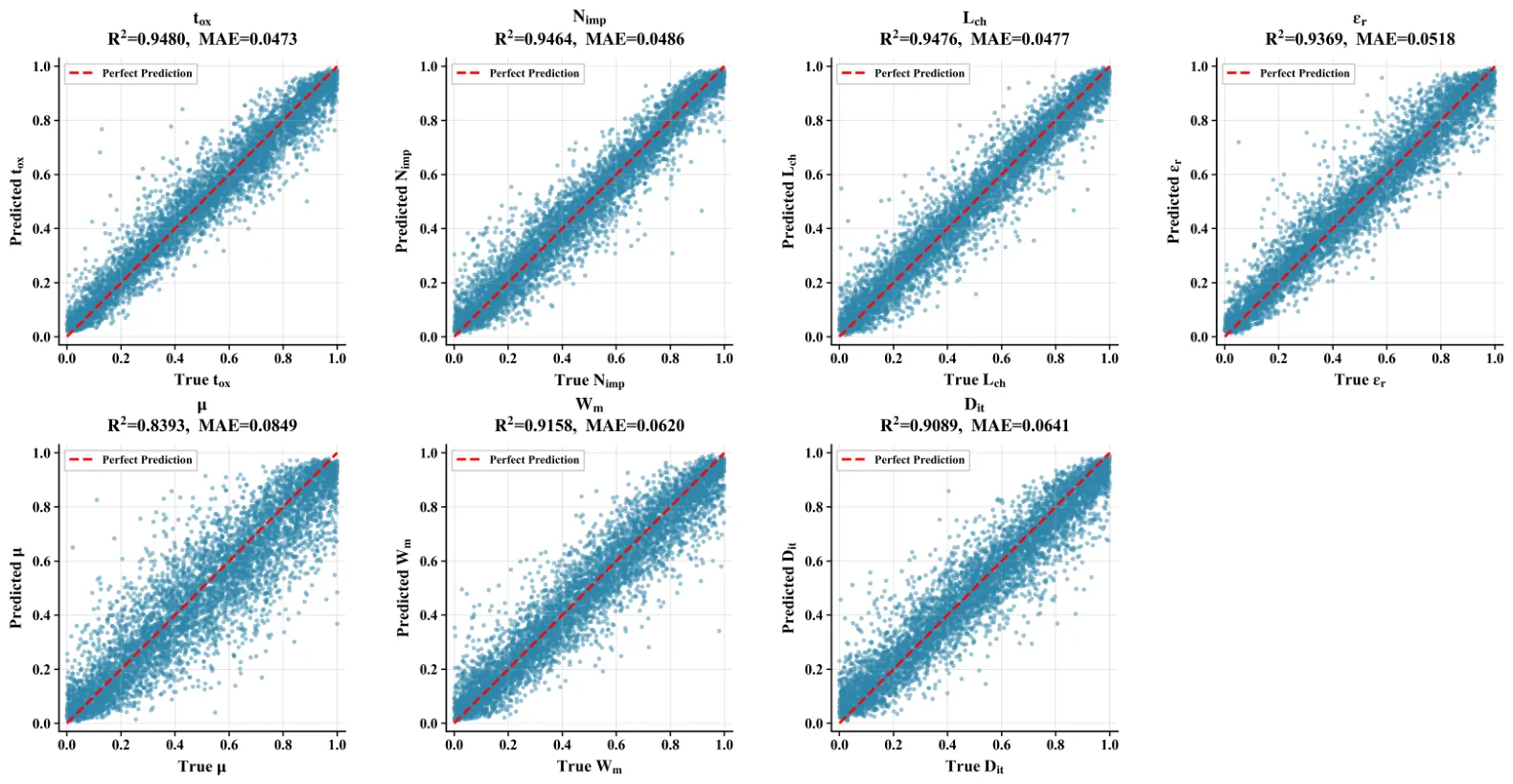
Scatter plots showing the predicted device parameters by the inverse S-cGAN model.

Figure 14 shows the agreement between the device parameters generated by the S-cGAN inverse design model and the corresponding parameters in the augmented dataset using scatter plots. The results show that most prediction points are distributed near the diagonal line. This indicates that the model can effectively capture the complex nonlinear mapping between target performance and device structural parameters. For mobility, although the inverse-prediction $R^{2}$ is relatively low, it remains within a reasonable range. As demonstrated by the SHAP analysis in Section 4.1, the initial mobility makes a relatively small contribution to device performance within the design range considered in this work. Therefore, its prediction deviation has only a limited effect on the overall design results. Nevertheless, under extreme conditions such as low mobility, mobility can significantly affect carrier transport. Retaining this parameter is therefore necessary to preserve the physical completeness of the design space and the physical plausibility of the inverse-design results.

Regarding the “one-to-many” nature of the inverse-design problem, this work primarily selects representative parameter combinations that satisfy the target-performance requirements for TCAD validation. In addition, the introduction of random noise into the inverse S-cGAN provides the possibility of exploring multiple solutions. For the same target performance, repeated random sampling can be used to generate multiple candidate parameter combinations, which can subsequently be screened and validated using the surrogate model and TCAD simulations according to their performance and design trade-offs. In the inverse-design process, the allowable ranges of the design parameters are defined according to the calibrated TCAD model and physical plausibility, ensuring that the generated results satisfy practical manufacturing and physical constraints. Within these constraints, the model can perform limited extrapolation in the vicinity of the calibrated design space. However, all generated results must remain physically admissible and be validated through TCAD simulations. The current work does not explicitly account for process variations during fabrication. In future work, random perturbations will be introduced into key process parameters to further evaluate the effects of process variability on device performance and the robustness of inverse design.

However, the performance labels in the augmented dataset are obtained from the forward performance prediction model. Therefore, certain prediction errors are inevitably introduced. If these performance labels are directly used as the reference targets, the errors may accumulate through the forward and inverse design process. This may reduce the physical reliability of the final design results. To address this issue, 200 new samples are selected for independent model validation. The device parameters generated by the inverse design model are fed back into the calibrated TCAD simulation model. The corresponding TCAD-simulated electrical performance is then obtained. The TCAD results are compared with the target performance to evaluate the physical consistency of the inverse design results.

Table 7 summarizes the errors between the target performance and the electrical performance obtained from TCAD validation of the inversely designed device parameters. The results show that the errors of all performance metrics are within an acceptable range, with an average MAE of 0.0464 and an average MAPE of 5.46%. This indicates that the inverse design method can accurately adjust the device structural parameters, enabling the predicted results to closely approach the target values. However, the MAPE values of SS and the $I_{d}$–$V_{g}$ curve are slightly higher. This is related to the higher prediction difficulty of the forward model for SS and subthreshold-region current. It is also affected by error accumulation in the inverse design process. Nevertheless, these errors remain within a reasonable range for engineering design. They do not affect the overall effectiveness of the proposed design method.

**Figure 15:**
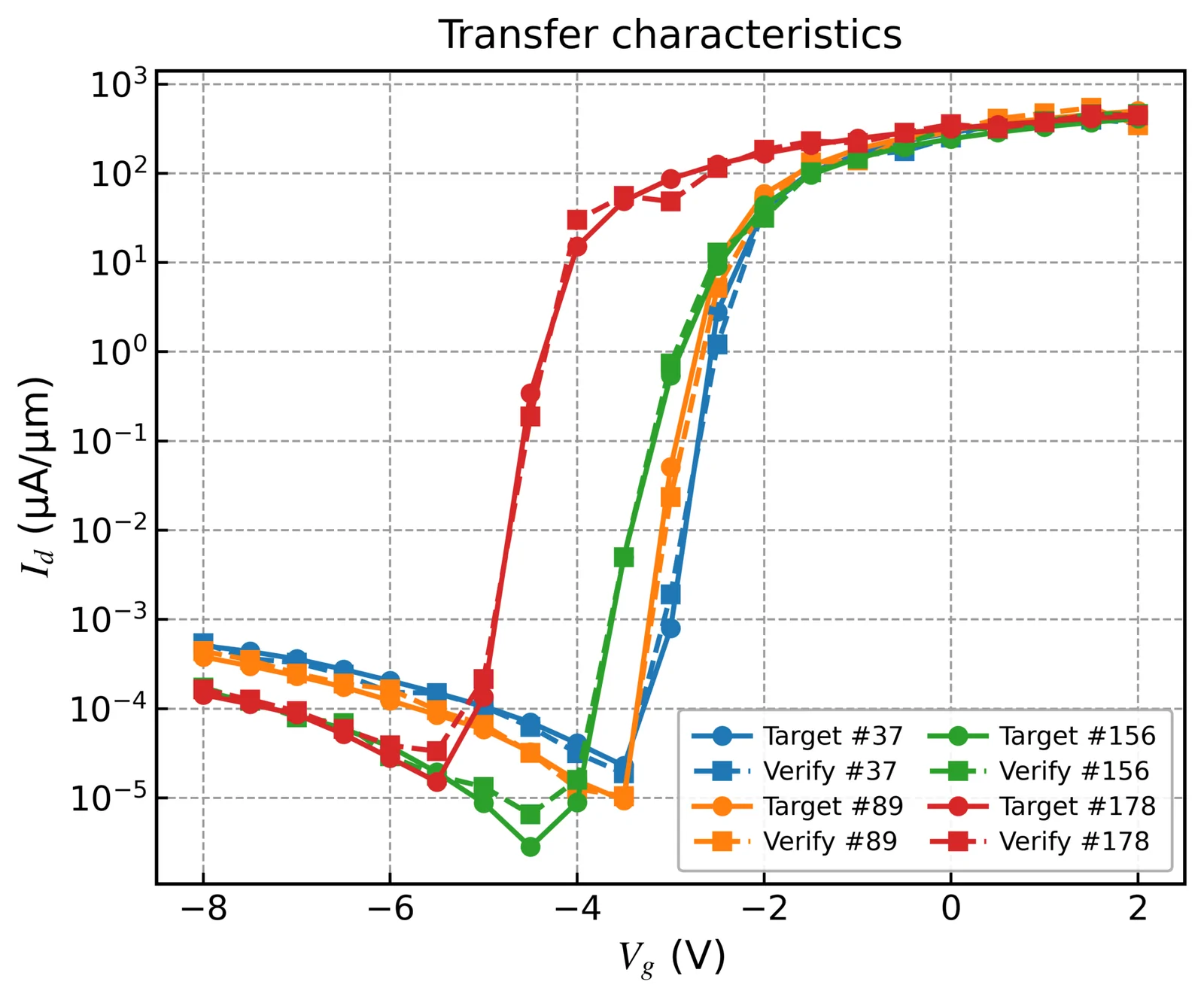
Target and TCAD-verified transfer characteristics of monolayer MoS_2_ FETs.

Figure 15 shows the electrical behavior of the inversely designed devices after TCAD validation in the form of transfer characteristic curves. It can be seen that, after the device parameters generated by the inverse design model are fed into the TCAD simulations, the resulting transfer characteristic curves agree well with the corresponding target-performance curves. These results indicate that the proposed method can accurately reproduce the key switching characteristics and threshold behavior of the target devices. These results strongly verify the physical reliability and engineering practicality of the proposed forward–inverse co-design framework.

### 4.3. Computational Efficiency Analysis

Table 8 summarizes the measured computational time required at each stage of the proposed framework. Generating the TCAD dataset containing 300 samples requires approximately 125–250 h and represents the most computationally expensive one-time stage of the framework, reflecting the high cost of data acquisition in small-sample modeling scenarios. In addition, training the forward and inverse models requires a total of 35.27 h. This training cost is also incurred only once and can be amortized over multiple subsequent design tasks. After training, each model inference requires less than 50 ms and is therefore negligible compared with the time required for TCAD simulation and model training. Thus, the computational cost of the proposed framework is primarily concentrated in the two one-time stages of TCAD dataset generation and model training, whereas the marginal cost of inference is almost negligible. Consequently, the framework is more suitable as an efficient tool for large-scale and high-frequency design iterations than for a one-off device-design task with a single target.

To quantify the application scenarios in which the proposed framework provides an overall computational advantage, an effective speedup ratio η is introduced. It is defined as the ratio of the total computational time required by conventional TCAD simulations to that required by the proposed framework:

(10) $$\eta =\frac{NT_{\mathrm{TCAD}}}{300T_{\mathrm{TCAD}}+T_{\mathrm{Train}}+NT_{\mathrm{Infer}}},$$

where *N* denotes the number of required TCAD simulation tasks, $T_{\mathrm{TCAD}}$ is the computational time required for a single TCAD simulation, $T_{\mathrm{Train}}$ is the total model-training time, and $T_{\mathrm{Infer}}$ is the time required for a single model inference. Because the inference time is less than 50 ms and is negligible relative to the other computational costs, Equation (10) can be simplified as

(11) $$\eta \approx \frac{NT_{\mathrm{TCAD}}}{300T_{\mathrm{TCAD}}+T_{\mathrm{Train}}}.$$

When η≤1, direct TCAD simulation is more computationally economical, whereas the proposed framework begins to exhibit an overall computational advantage when η>1.

In addition, an inference speedup ratio α is introduced to quantitatively evaluate the acceleration achieved by the trained model:

(12) $$\alpha \geq \frac{T_{\mathrm{TCAD},\min}}{T_{\mathrm{Infer},\max}},$$

where $T_{\mathrm{TCAD},\min}$ denotes the minimum time required for a single TCAD simulation, and $T_{\mathrm{Infer},\max}$ denotes the maximum model-inference time. As shown in Table 8, a single TCAD simulation of a monolayer MoS_2_ FET requires approximately 25–50 min, depending on the device structure, whereas a single prediction by the trained model can be completed within 50 ms. Therefore, the inference speedup relative to a single TCAD simulation satisfies $\alpha \geq 3\times 10^{4}$, corresponding to an improvement of approximately four orders of magnitude in computational efficiency. It should be emphasized that this speedup ratio applies only to a single inference after model training and does not include the one-time computational costs of TCAD dataset construction and model training.

**Figure 16:**
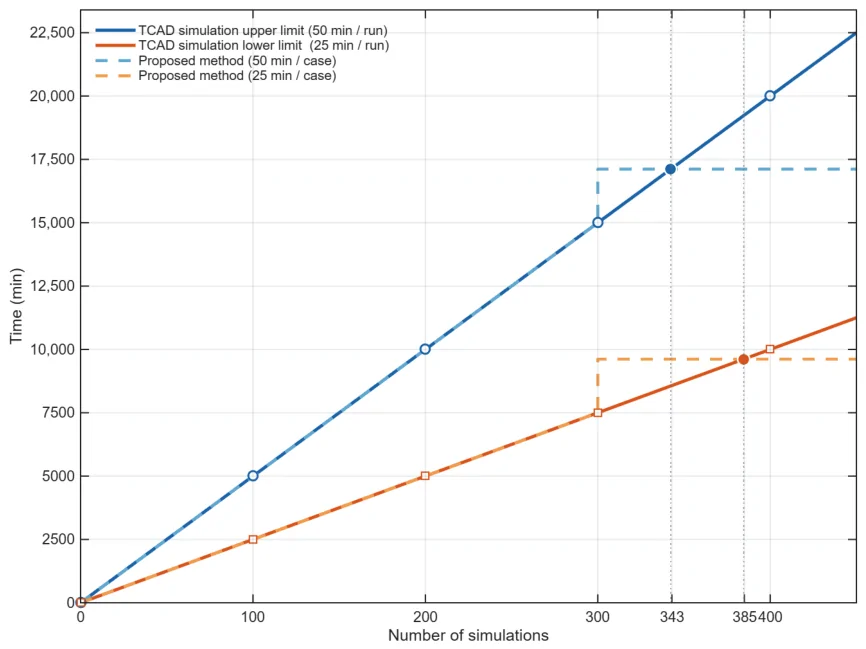
Cumulative computational time comparison of TCAD simulation and the proposed framework.

Figure 16 illustrates the cumulative computational time required by conventional TCAD simulations and the proposed framework as the number of simulation tasks increases. The cumulative computational cost of conventional TCAD simulation increases approximately linearly with the number of simulations. In contrast, the computational cost of the proposed framework is concentrated primarily in the initial generation of 300 TCAD samples and model training. Once training is complete, the inference time of less than 50 ms per task is almost negligible. For monolayer MoS_2_ FET simulation tasks, when a single TCAD simulation requires 25 min, the two approaches reach the break-even point at approximately 385 simulation tasks. When the time required for a single TCAD simulation increases to 50 min, the break-even point decreases to approximately 342 tasks. Thus, as the computational cost of an individual TCAD simulation increases, fewer design tasks are required for the proposed framework to recover its one-time costs associated with dataset generation and model training. Before the break-even point, direct TCAD simulation is more economical. Beyond this point, the efficiency advantage of the proposed framework increases progressively with the number of design tasks, making it more suitable for large-scale parameter sweeps, device screening, and high-frequency design iterations.

The computational advantage of the proposed framework becomes even more significant in inverse-design tasks. Conventional optimization methods, such as genetic algorithms (GA), particle swarm optimization (PSO), and differential evolution (DE), typically require repeated TCAD simulations to evaluate a large number of candidate structures. For the seven-dimensional design space considered in this work, assuming that approximately $10^{3}$–$10^{4}$ TCAD calls are required for convergence, the total computational time is approximately 416.67–8333.33 h when each simulation requires 25–50 min. In comparison, after accounting for the one-time costs of data acquisition and model training, the total computational cost of the proposed framework is approximately 160.27–285.27 h. The proposed framework can therefore save approximately 256.40–8048.06 h compared with conventional optimization methods. Moreover, the trained model can be reused for inverse-design tasks involving different target-performance specifications. As the number of design tasks increases, the one-time computational cost is further amortized, making the computational advantage increasingly significant.

As device structures and physical models become more complex, TCAD simulations generally require finer meshes, more sophisticated physical models, and a greater number of nonlinear iterations, further increasing the computational time of each simulation. Under such conditions, the one-time costs of dataset construction and model training can be amortized over fewer design tasks, and the computational-efficiency advantage of the proposed framework becomes more pronounced. Therefore, the proposed framework has greater application potential in computationally intensive scenarios involving complex devices, multiphysics coupling, and large-scale parameter optimization.

It should be noted that all computational times reported in this work were measured on a server equipped with two Intel Xeon Platinum 8570 CPUs. No discrete graphics card or GPU acceleration was used during the calculations. Both the TCAD simulations and the training and inference of the machine learning models were performed entirely on CPUs.

## 5. Conclusions

This paper proposes an efficient automated design framework for 2D TMD FETs under small-sample conditions. A monolayer MoS_2_ FET is used as a case study for systematic validation. By constructing a unified framework that integrates device design, physics-based simulation, performance prediction, and inverse design, the proposed method establishes a two-way prediction between device structural parameters and target electrical performance. It avoids the dependence on computationally intensive TCAD simulations and manual parameter tuning. It also enables efficient and automated design of the key structural parameters of monolayer MoS_2_ FETs. In terms of computational efficiency, even after accounting for the costs of TCAD data generation and model training, the proposed framework retains a significant computational advantage in large-scale parameter sweeps and high-frequency design iterations. In addition, the proposed method shows good portability and scalability. It can be further extended to the design optimization of other emerging semiconductor devices. The proposed method is primarily validated using an experimentally calibrated TCAD model, while the corresponding devices have not yet been fabricated or experimentally characterized. This represents one of the main limitations of the current work. In future work, representative inverse-designed monolayer MoS_2_ FETs will be selected for fabrication, and their electrical performance will be experimentally evaluated. The observed process variations and measurement results will subsequently be fed back into the TCAD and machine learning models to progressively establish an experimental closed-loop workflow encompassing design, fabrication, testing, and model refinement, thereby further improving the engineering feasibility and reliability of the proposed framework.

## Figures and Tables

**Table 1:** Parameters of the calibrated TCAD model for the monolayer MoS_2_ FET.

Category	Parameter	Value	Unit
Geometry and materials	Density-of-states model	Subband 1 + 2	–
	$T_{\mathrm{vdW}}$	0.27	nm
	$T_{{\mathrm{MoS}}_{2}}$	0.3	nm
	$T_{{\mathrm{HfO}}_{2}}$	8.5	nm
	EOT	2.65	nm
	HfO_2_ relative permittivity	12.5	–
	MoS_2_ relative permittivity	11.7	–
	vdW-gap relative permittivity	1.0	–
	vdW-gap bandgap	9	eV
	MoS_2_ bandgap	1.8	eV
Transport and contact	S/D doping concentration	$1.6\times 10^{19}$	cm^−3^
	vdW-gap $m_{t}$	0.93	–
	$C_{\exp}$	0.5	–
	$N_{c,\exp}$	1.0	–
	η	$5.82\times 10^{30}$	–
	$v_{\mathrm{sat}}$	$3\times 10^{5}$	cm/s
	Schottky-barrier height	0	eV
	$\mu_{\max}$	55	cm^2^/(V·s)
	Sb resistivity	$1.1\times 10^{-5}$	Ω·cm
	Gate work function	4.2	eV
	S/D contact work function	4.4	eV
Interface traps	Uniform-distribution σ	1.8	eV
	Gaussian-distribution σ	0.2	eV
	Gaussian trap energy midpoint relative to the conduction band	0.7	eV

**Table 2:** Design parameter ranges of monolayer MoS_2_ FETs.

Parameter	Physical Meaning	Range	Unit
$L_{\mathrm{ch}}$	Channel length	0.02–0.1	μm
$t_{\mathrm{ox}}$	Gate oxide thickness	4–10	nm
$W_{m}$	Metal work function	4.3–5.0	eV
$N_{\mathrm{imp}}$	Doping concentration	1–10	$10^{12}\;{\mathrm{cm}}^{-2}$
$\varepsilon_{r}$	Relative permittivity	8–18	–
μ	Mobility	50–100	cm^2^/(V·s)
$D_{\mathrm{it}}$	Trap density	1–10	${\mathrm{eV}}^{-1}\;{\mathrm{cm}}^{-2}$

**Table 3:** Baseline forward-regression models and key hyperparameter settings.

Algorithm	Core Hyperparameters	Configuration
ANN	learning rate, reconstruction weight	$1\times 10^{-4}$; 10
RF	Number of trees	200
GB	Number of estimators, learning rate, maximum depth	200; 0.05; 4
Ridge	Regularization coefficient	1.0
SVR	Penalty parameter, insensitive loss	10.0; 0.05

**Table 4:** Ablation study of the discriminator under different training sample sizes.

Training Samples	$R^{2}$ (S-cGAN with Discriminator)	$R^{2}$ (ANN Without Discriminator)
50	0.7388	0.7691
100	0.8952	0.9132
150	0.8798	0.8483
200	0.9271	0.9146
250	0.9437	0.9390
300	0.9503	0.9469

**Table 5:** Prediction accuracy for key electrical performances and $I_{d}$–$V_{g}$ characteristics.

Performance	$R^{2}$	MAE	MSE	MAPE (%)
$V_{\mathrm{th}}$	0.9844	0.0223	0.0008	6.67
$I_{\mathrm{on}}$	0.9818	0.0194	0.0006	1.48
$I_{\mathrm{off}}$	0.8981	0.0472	0.0046	1.60
SS	0.9036	0.0465	0.0040	7.31
$I_{d}$–$V_{g}$ curve	0.9836	0.0232	0.0020	7.80

**Table 6:** Statistical summary of $R^{2}$ values obtained from 1000 bootstrap iterations.

Output	Mean	Standard Deviation	Median	95% Confidence Interval
$V_{\mathrm{th}}$	0.9868	0.0022	0.9871	[0.9818,0.9904]
$I_{\mathrm{on}}$	0.9476	0.0050	0.9478	[0.9369,0.9566]
$I_{\mathrm{off}}$	0.8792	0.0146	0.8811	[0.8471,0.9029]
SS	0.8471	0.0214	0.8489	[0.8002,0.8833]
$I_{d}$–$V_{g}$ curve	0.9878	0.0011	0.9879	[0.9855,0.9898]
Average	0.9297	0.0056	0.9300	[0.9183,0.9396]

**Table 7:** Errors between the target performance metrics and the TCAD-validated results.

Performance	MAE	MAPE (%)
$V_{\mathrm{th}}$	0.0245	5.15
$I_{\mathrm{on}}$	0.0495	2.52
$I_{\mathrm{off}}$	0.0543	2.51
SS	0.0754	8.22
$I_{d}$–$V_{g}$ curve	0.0282	8.92

**Table 8:** Computational cost comparison of TCAD simulation, S-cGAN prediction, and inverse design.

Stage	Time
Single TCAD Simulation	25–50 min (depending on the device structure)
S-cGAN Model Training	0.39 h
S-cGAN Model Single Prediction	<50 ms
Inverse Design Model Training	34.88 h
Inverse Design Model Single Prediction	<50 ms

## Data Availability

The data that support the findings of this study are available from the corresponding author upon reasonable request.
